# Routinely available perioperative variables and EHR-captured postoperative adverse-event indicators after adult abdominal surgery: A retrospective cohort study using MOVER EPIC

**DOI:** 10.1371/journal.pone.0350704

**Published:** 2026-10-21

**Authors:** Anqi Shi, Xinyue Cui, Hanling Zheng, Yifan Li

**Affiliations:** 1 Department of General Surgery, Traditional Chinese Medicine Hospital of Qingbaijiang Affiliated to Chengdu Medical College, Chengdu, China; 2 Department of International Research Collaboration, Sichuan Global Zhongsen Tanke Technology Co., Ltd., Chengdu, China; 3 Department of General Surgery, Sichuan Provincial First Veterans Hospital, Chengdu, China; 4 Faculty of Clinical Medical College, Chengdu Medical College, Chengdu, China; Mercedes-Benz Group AG, UNITED STATES OF AMERICA

## Abstract

**Background:**

Perioperative electronic health record (EHR) datasets are increasingly used to study postoperative morbidity, although database-recorded adverse-event indicators may not correspond to standardized complication endpoints. We characterized these indicators after adult abdominal surgery in MOVER EPIC and examined associations with routinely available perioperative variables.

**Methods:**

This retrospective cohort study identified adult abdominal surgical encounters from 2017–2022 through procedure-name screening and case-level deduplication. The primary outcome was a broad composite of EHR-captured postoperative adverse-event indicators recorded within the indexed perioperative encounter. The secondary outcome was a narrower composite of severe EHR-captured adverse-event indicators. Multivariable logistic regression was performed in the primary complete-case cohort, adjusting for age, sex group, ASA physical status, procedure group, and operating-room duration. Sensitivity analyses included ASA-excluded models, alternative duration specifications, first-encounter restriction, penalized regression, patient-level cluster-robust inference, and inverse probability weighting (IPW).

**Results:**

Among 5,679 encounters, the broad composite occurred in 166 (2.92%), the narrow severe composite in 95 (1.67%), and death in 23 (0.40%). In the primary complete-case cohort (n = 5,349; 147 broad events), ASA IV + was strongly associated with the broad composite compared with ASA III (adjusted OR 4.66, 95% CI 2.92–7.45; p < 0.001). Laparotomy/adhesiolysis was also associated with higher odds (OR 1.79, 95% CI 1.02–3.13; p = 0.041), whereas spline analyses suggested modest nonlinearity in the age association; operating-room duration was not independently associated. IPW estimates were materially similar, although the estimate for ASA I–II was less precise.

**Conclusions:**

Clinician-assigned ASA physical status was the most consistent correlate of EHR-captured postoperative adverse-event indicators. ASA may reflect comorbidity burden and perceived perioperative vulnerability rather than directly measuring physiological reserve. These findings should not be interpreted as standardized 30-day postoperative complication risks.

## Introduction

Adult abdominal surgery encompasses a heterogeneous range of procedures involving the gastrointestinal tract, hepatobiliary and pancreatic system, abdominal wall, and other intra-abdominal structures. Despite advances in surgical techniques, anesthesia management, and perioperative care, postoperative complications remain important determinants of surgical safety, recovery, and healthcare resource use. In the International Surgical Outcomes Study, 16.8% of adult patients undergoing elective inpatient surgery developed at least one postoperative complication, and these complications were associated with longer hospital stay, increased critical care use, and mortality following complications [1]. In abdominal surgery, particularly major or high-risk procedures, postoperative morbidity is unlikely to be explained by procedural factors alone. Evidence from studies of major abdominal surgery indicates that frailty and reduced physiological reserve are strongly associated with major morbidity and mortality [2], while perioperative cohort data suggest that American Society of Anesthesiologists (ASA) physical status, anemia, blood loss, and intraoperative hemodynamic events may also contribute to postoperative risk [3]. These findings support the need to examine simple, routinely available, and clinically interpretable risk signals in real-world adult abdominal surgery populations. Although such studies provide important context for the clinical burden of postoperative complications, their event rates should not be directly compared with EHR-derived perioperative complication indicators that lack a standardized 30-day ascertainment window.

Previous research has substantially advanced perioperative risk assessment. Large surgical databases have enabled the development of risk calculators for individualized preoperative risk estimation and shared decision-making [4]. The Estimation of Physiologic Ability and Surgical Stress (E-PASS) framework further emphasizes that postoperative outcomes reflect the interaction between preoperative physiological capacity and surgical stress, rather than surgical exposure alone [5]. More recently, electronic health record and perioperative database studies have expanded the use of machine-learning methods for postoperative complication prediction and surveillance. Some models integrate preoperative and intraoperative data to predict specific postoperative complications, whereas others use postoperative EHR data to support automated complication identification and surveillance [6,7]. However, these tools often prioritize prediction performance or automated event detection and may not directly clarify the relative contribution of simple clinical variables in heterogeneous abdominal surgery cohorts. It therefore remains important to determine whether routinely available perioperative variables retain independent associations with postoperative complications after broad procedural differences are considered.

The Medical Informatics Operating Room Vitals and Events Repository (MOVER) is a public perioperative data resource containing electronic health record data and high-fidelity intraoperative physiological waveforms from adult surgical patients at the University of California, Irvine Medical Center [8]. In the present study, we used the EPIC electronic health record component of MOVER rather than high-frequency waveform data. This study was not designed to develop a new prediction model or to estimate standardized 30-day postoperative complication risk. Instead, it aimed to characterize EHR-captured postoperative adverse-event indicators in adult abdominal surgery encounters and to examine how routinely available perioperative variables, including ASA physical status, procedure group, sex, age, and operating-room duration, relate to these database-captured morbidity signals. By explicitly distinguishing EHR-derived adverse-event indicators from validated postoperative complication endpoints, this analysis provides a transparent description of how simple clinical variables behave in an exploratory public perioperative EHR dataset.

## Methods

### Study design and data source

This retrospective cohort study used electronic health record data from the Medical Informatics Operating Room Vitals and Events Repository (MOVER), a publicly available perioperative database for adult surgical patients [8]. The present analysis was based on the EPIC component of MOVER, which contains perioperative records for adult patients treated at the University of California, Irvine Medical Center, a single-center academic hospital in Orange County, California, USA, between 2017 and 2022. The database includes routinely collected perioperative information, including demographic characteristics, procedure records, anesthesia-related variables, hospitalization data, and postoperative outcome data. In the current study, these data were used to construct an abdominal surgery cohort and to evaluate EHR-recorded postoperative adverse-event indicators and their associated perioperative factors. The unit of analysis was the surgical encounter, with LOG_ID used as the case-level identifier. Before cohort construction, the downloaded MOVER EPIC source files were checked using the database-provided file-verification procedure to confirm data integrity. Only verified source files were used for extraction, cleaning, and analysis.

### Cohort identification

The study cohort was constructed from case-level perioperative records in the EPIC component of MOVER. Each surgical encounter was treated as the analytic unit, and LOG_ID was used as the case-level identifier. Records were initially screened against a prespecified allowlist of abdominal surgery procedure names, and non-target procedures were excluded to define the abdominal surgery cohort.

To address instances where multiple raw records corresponded to the same LOG_ID, case-level deduplication was performed to ensure each LOG_ID contributed only one analytic record. When duplicates were present, the record with the most complete information on procedure name, surgery date, and operating-room timing was retained. Following age derivation, the cohort was restricted to adult surgical encounters, defined as age 18 years or older at the time of surgery.

The deduplicated adult analytic cohort was subsequently used for descriptive analyses and outcome frequency summaries. All supplementary tables cited below are provided in S1 File. Within this file, Supplementary Table S1 in S1 File details the deduplication rules, while Supplementary Table S2 in S1 File summarizes the procedure-name inclusion and exclusion criteria. The overall cohort derivation process, including the primary complete-case regression cohort and the larger sensitivity-analysis cohort excluding ASA, is depicted in Fig 1. This study was reported in accordance with the Strengthening the Reporting of Observational Studies in Epidemiology (STROBE) statement for cohort studies in supplementary Table S18 in S1 File.

**Fig 1:**
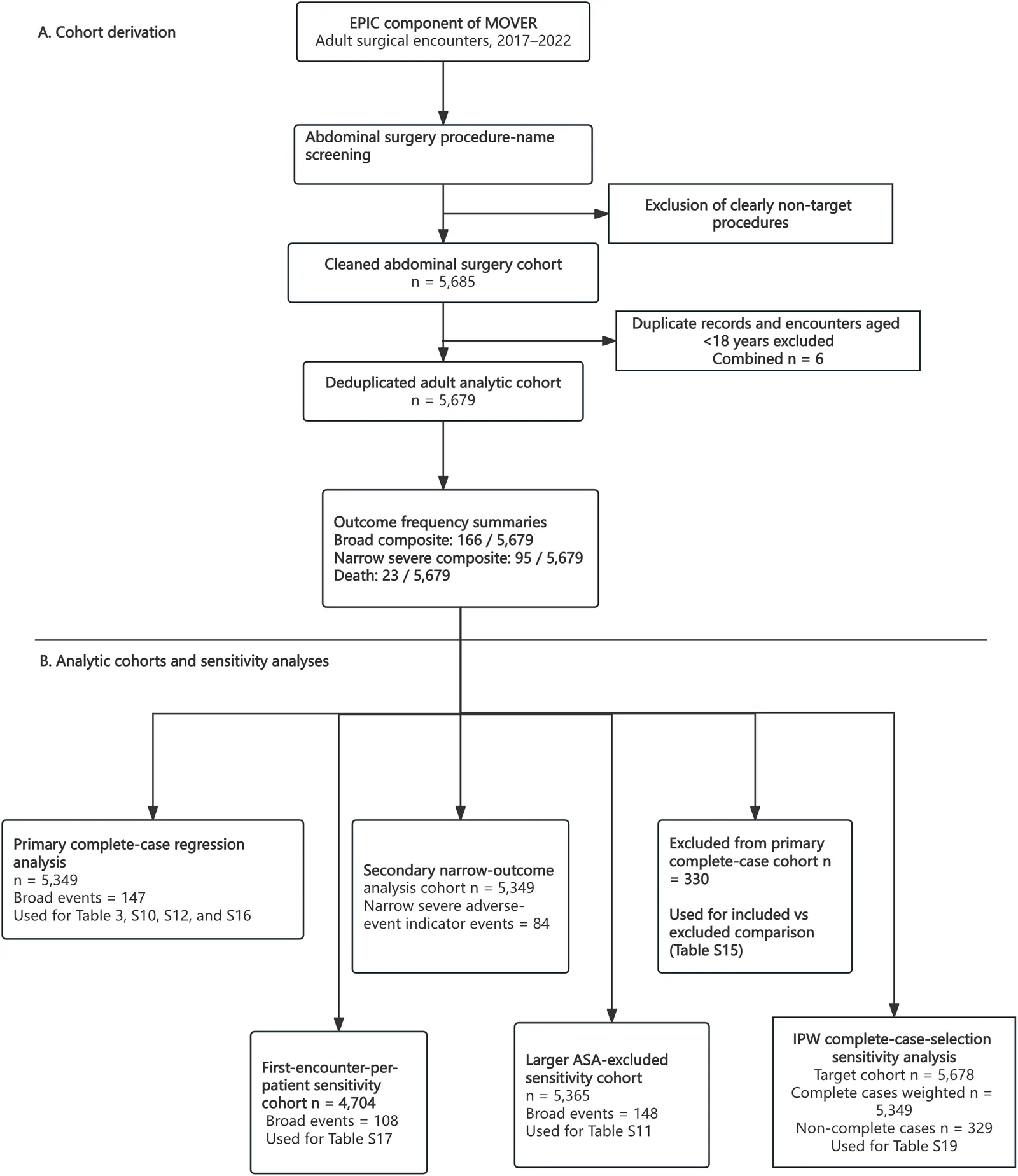
Cohort derivation, outcome frequencies, primary and secondary analytic cohorts, and sensitivity analyses, including inverse probability weighting for complete-case selection. Note: The deduplicated adult analytic cohort (n = 5,679) was used for outcome frequency summaries and available-case descriptive comparisons. The primary complete-case cohort (n = 5,349; broad composite events = 147) required non-missing values for the primary outcome, age, sex group, ASA group, procedure group, and operating-room duration. This cohort was used for the primary multivariable model, primary univariable analyses, the same-sample ASA-excluded model, the narrow-outcome models (narrow composite events = 84), and the categorized operating-room duration sensitivity analysis. The larger ASA-excluded sensitivity cohort (n = 5,365; broad composite events = 148) excluded ASA from the complete-case requirement. The first-encounter-per-patient sensitivity cohort retained the earliest eligible encounter per patient from the primary complete-case cohort (n = 4,704; broad composite events = 108). The IPW target cohort included 5,678 encounters with Female or Male sex coding; 5,349 were complete cases and 329 were non-complete cases.

### Outcome definitions

The outcomes in this study consisted of operational electronic health record (EHR)-derived indicators based on database-recorded postoperative adverse-event fields. These variables did not represent externally adjudicated postoperative complications and lacked a uniform 30-day postoperative ascertainment window. Therefore, all outcomes were interpreted as database-captured postoperative morbidity indicators within the indexed perioperative encounter and were not considered validated postoperative complication endpoints.

The primary outcome was a broad composite of EHR-captured postoperative adverse-event indicators, defined by the binary variable comp_system_broad. This variable was coded as 1 if at least one database-recorded postoperative adverse-event category meeting the prespecified broad definition was present for the indexed surgical encounter; otherwise, it was coded as 0.

The broad composite included higher-level database adverse-event categories, including cardiovascular, respiratory, airway, neurological, metabolic, injury or infection, and death-related events. This outcome was designed to maximize the capture of postoperative morbidity signals recorded in the database and was not intended to represent a severity-graded or clinically adjudicated complication endpoint.

The secondary outcome was a narrow severe EHR-captured adverse-event indicator composite, defined by the binary variable comp_severe_narrow. This variable was coded as 1 if at least one database-recorded event met the prespecified severe adverse-event criteria, and 0 otherwise. In contrast to the broad composite, the narrow composite was restricted to event-level indicators that more directly reflected major physiological deterioration or unplanned escalation of postoperative care, including acute hemodynamic instability, clinically relevant respiratory compromise, cardiac arrest or arrhythmia, aspiration, unplanned postoperative ventilatory support, reintubation, unplanned intensive care unit admission recorded as a postoperative adverse-event item, and death. This narrow outcome therefore served as a sensitivity-oriented measure of more serious database-recorded morbidity signals and was not intended to represent a formal Clavien-Dindo grade.

Thus, the broad composite prioritized capture of database-recorded postoperative morbidity signals, whereas the narrow composite prioritized specificity for more serious or escalation-related events. Component-specific inclusion and exclusion rationales are provided in Supplementary Table S4a in S1 File.

Death was included as a component of both composite outcomes when recorded in the postoperative adverse-event data and was also reported separately as a secondary outcome using the death_flag variable. This separate reporting was implemented to ensure transparency and should not be interpreted as duplicate counting within the composite outcomes. Each composite outcome was coded at the case level; therefore, encounters with multiple component events contributed only once to the corresponding composite outcome.

ICU-related variables were handled according to their source and interpretation. Unplanned ICU admission recorded as a postoperative complication was included in the narrow severe EHR-captured adverse-event indicator composite because it indicated an unplanned postoperative escalation event. In contrast, administrative ICU indicators, such as ICU_ADMIN_FLAG or icu_admin_true, were not considered primary or secondary outcomes because they may represent planned ICU use, monitoring practices, or broader resource utilization rather than adjudicated postoperative complications. Detailed outcome definitions are provided in Supplementary Table S3 in S1 File, and the component events of the composite outcomes are listed in Supplementary Table S4a in S1 File. Component-level frequencies for the broad and narrow EHR-captured adverse-event indicator composites in the final adult analytic cohort are provided in Supplementary Table S4b in S1 File.

### Candidate predictors and covariates

Candidate predictors and covariates were prespecified based on clinical relevance and the perioperative information available in the EPIC component of MOVER. These variables were grouped into four domains: demographic characteristics, baseline clinical status, perioperative management, and procedure-related factors. Demographic variables included age and sex. Baseline clinical status was primarily represented by the American Society of Anesthesiologists (ASA) physical status classification. Perioperative variables included operating-room duration, anesthesia duration, primary anesthetic technique, and patient class. Procedure-related information was derived from the indexed procedure to represent surgical heterogeneity.

In the primary multivariable model, priority was assigned to variables with clear clinical interpretability, appropriate temporal relationship to the postoperative outcome, and acceptable data completeness. The prespecified covariates included age, sex group, ASA group, procedure group, and operating-room duration. Age and operating-room duration were modeled as continuous variables. Operating-room duration was retained as a clinically interpretable proxy for procedural exposure and operative complexity.

To enhance interpretability and reduce data sparsity, the original procedure categories were consolidated into four clinically coherent groups: low-complexity common abdominal procedures included appendectomy, cholecystectomy, and hernia repair; intestinal resection/stoma procedures included colorectal resection, small-bowel resection, and stoma surgery; upper abdominal major surgery included gastrectomy, pancreatic surgery, and splenic procedures that had initially been retained in the residual “Other” category; and laparotomy/adhesiolysis was retained as a separate analytic group. This grouping was intended to preserve clinically interpretable differences in operative anatomy and complexity while avoiding excessively sparse procedure categories in regression models. The full mapping of the original procedure categories to the four analytic groups is provided in Supplementary Table S6 in S1 File, and the clinical definitions and representative procedures are summarized in Supplementary Table S20 in S1 File.

Anesthesia duration was retained for descriptive and supplementary characterization but was excluded from the primary model due to its conceptual overlap with operating-room duration. Hospital length of stay was summarized descriptively but not included as a predictor in regression models, as it was considered an outcome-related postoperative characteristic rather than a baseline or intraoperative covariate. Other variables, such as patient class and primary anesthetic technique, were retained for descriptive or supplementary purposes but were not included in the final primary model due to model stability, interpretability, completeness, or other factors. Detailed variable definitions, source fields, derivation rules, and analytical roles are provided in Supplementary Table S5 in S1 File.

### Data cleaning and variable derivation

Following case-level deduplication and cohort construction, the raw perioperative records underwent further cleaning and variable derivation to produce the final analytic dataset for descriptive analyses and regression modeling. This process included timestamp parsing, derivation of continuous variables, standardization of categorical variables, handling of implausible values, exclusion of non-adult encounters, and consolidation of sparse categories where appropriate.

Date and time fields were standardized to facilitate the derivation of time-dependent variables. Surgery date, operating-room entry and exit times, and anesthesia start and stop times were parsed and harmonized from the original electronic health record fields. In the MOVER EPIC source files used for this study, the field labeled BIRTH_DATE contained patient age in years rather than a calendar birth date. Age was therefore converted directly to a numeric value and was not calculated as the interval between birth date and surgery date. The cohort was restricted to adult surgical encounters involving patients aged 18 years or older. Operating-room duration was defined as the interval between operating-room exit and entry, and anesthesia duration as the interval between anesthesia stop and start. Implausible or clearly unrealistic values were recoded as missing prior to analysis according to prespecified plausibility rules.

Categorical variables were standardized and regrouped as necessary for analysis. Sex was harmonized into standard analytical categories, and the single record coded as “Other” was excluded from male-versus-female comparisons and from regression models involving sex group due to sparse data. ASA physical status was derived from the original numeric ASA_RATING_C field to ensure consistent classification across records. ASA codes 1 and 2 were grouped as ASA I–II, code 3 as ASA III, and codes 4–6 as ASA IV + . The numeric coding was used as the primary reference because text-based ASA descriptions showed inconsistent formatting across source records. The resulting ASA categories were used in all primary and sensitivity analyses. Procedure information derived from the indexed operation was used both for cohort construction and for assignment to the four analytic procedure groups described above, with detailed mappings provided in Supplementary Tables S6 and S20 in S1 File. Study outcomes were standardized as case-level binary variables, including the broad EHR-captured postoperative adverse-event indicator composite, the narrow severe EHR-captured adverse-event indicator composite, death, and exploratory ICU-related indicators. Continuous variables were converted to numeric values and expressed in hours, as appropriate for modeling. Detailed cleaning rules, derivation procedures, and variable mappings are summarized in Supplementary Tables S6 and S7 in S1 File.

### Missing data handling

Missingness was assessed following case-level deduplication, adult cohort restriction, outcome standardization, and variable derivation. For prespecified analysis variables, the number and proportion of missing observations were quantified in the deduplicated adult analytic cohort. Because this study used routinely collected perioperative electronic health record data rather than prospectively curated research variables, missingness was considered inherent to the real-world data structure. No formal imputation procedure was applied. Detailed missingness summaries are provided in Supplementary Table S8a in S1 File.

Descriptive analyses and outcome frequency summaries were conducted using the deduplicated adult analytic cohort. For baseline comparisons by primary outcome status, variable-specific available-case denominators were used, resulting in differing effective sample sizes across variables. For sex-specific comparisons, the single record coded as “Other” was excluded because it was not analytically meaningful for the male-versus-female comparison.

The primary complete-case adult cohort included encounters with non-missing values for the primary outcome and all prespecified covariates: age, sex group, ASA group, procedure group, and operating-room duration. Following correction of the ASA classification using the original numeric ASA_RATING_C field, 5,349 of 5,679 encounters (94.2%) were included in the primary complete-case regression analyses, whereas 330 encounters (5.8%) were excluded. Missing ASA group values were present in 291 encounters (5.1%), and operating-room duration was missing in 314 encounters (5.5%), with substantial overlap between these sources of missingness; 275 encounters had both values missing. Age and procedure group were complete after cohort derivation, and one encounter with a non-binary sex category was not included in the regression model. To assess the influence of ASA-related complete-case exclusion, a larger sensitivity-analysis cohort was constructed by excluding ASA from the complete-case requirement. This cohort required non-missing values for the primary outcome, age, sex group, procedure group, and operating-room duration and included 5,365 encounters.

The secondary analysis using the narrow severe EHR-captured adverse-event indicator composite was conducted within the same primary complete-case adult cohort to maintain consistency in the covariate structure and sample definition across the primary and secondary outcome models. Model-specific analytic cohort definitions, sample sizes, event counts, and event rates are summarized in Supplementary Table S8b in S1 File.

### Statistical analysis

All analyses were conducted following case-level deduplication, adult cohort restriction, variable derivation, and outcome standardization. Baseline characteristics were summarized for the deduplicated adult analytic cohort and compared by primary outcome status. Categorical variables were summarized as counts and percentages and compared using chi-square tests. Continuous variables were summarized using medians and interquartile ranges and compared using Mann–Whitney U tests. Descriptive comparisons were based on available-case data, with variable-specific denominators reported in the tables.

The primary inferential analysis used the broad EHR-captured postoperative adverse-event indicator composite as the dependent variable. Univariable and multivariable logistic regression models were fitted in the primary complete-case adult cohort defined for the primary model covariates. The prespecified covariates for the primary multivariable model were age, sex group, ASA group, procedure group, and operating-room duration. Age and operating-room duration were modeled as continuous variables. Adjusted associations were reported as odds ratios (ORs) with 95% confidence intervals (CIs) and p values. Univariable logistic regression results for the primary outcome were reported using the same primary complete-case adult cohort to allow direct comparison between crude and adjusted associations.

Two ASA-excluded sensitivity models were used for complementary purposes. First, an ASA-excluded model was fitted in the larger complete-case cohort (n = 5,365) to assess the influence of ASA-related missingness. Second, an ASA-excluded model was fitted within the same primary complete-case cohort used for the main model (n = 5,349) to isolate the effect of removing ASA adjustment while holding the analytic sample constant.

To evaluate potential complete-case selection bias, encounters included in the primary complete-case cohort were compared with those excluded from the primary model using age, sex group, procedure group, the broad EHR-captured postoperative adverse-event indicator composite, and hospital length of stay.

To assess the linearity assumption for the continuous covariates, age and operating-room duration were each additionally modeled using centered restricted cubic spline terms with three degrees of freedom and a centering constraint, and the spline models were compared with the corresponding linear specifications using likelihood-ratio tests. Operating-room duration was also examined in a clinically interpretable categorical sensitivity analysis using <2 h, 2–4 h, and >4 h categories. Because the surgical encounter was the analytic unit and repeated encounters from the same patient could occur, two complementary sensitivity analyses were performed: the primary model was refitted using patient-level cluster-robust standard errors based on the patient identifier, and a first-encounter-per-patient analysis was performed by retaining only the earliest eligible abdominal surgical encounter for each patient and refitting the primary multivariable model.

A secondary analysis was conducted using the narrow severe EHR-captured adverse-event indicator composite as the dependent variable. Univariable and multivariable logistic regression models were fitted using the same covariate structure as the primary model, including age, sex group, ASA group, procedure group, and operating-room duration. Because the number of narrow severe-complication events was lower than that of the broad composite outcome, this secondary analysis was interpreted as supportive rather than definitive.

Multicollinearity among retained covariates was assessed before multivariable modeling, and no evidence of problematic collinearity was observed. Model stability was further evaluated by examining events per estimated parameter, convergence behavior, potential separation, and variance inflation factors. Because sparse events may influence maximum-likelihood logistic regression estimates, Firth penalized logistic regression was performed as a sensitivity analysis for the primary and secondary outcomes. In addition, patient-level clustering was considered using cluster-robust standard errors because multiple surgical encounters from the same patient could occur.

To further assess potential complete-case selection bias, an inverse-probability-weighted sensitivity analysis was performed among encounters eligible for the binary sex contrast (n = 5,678). A logistic regression model estimated the probability of inclusion in the primary complete-case cohort using variables available across this target cohort that could be associated with completeness or the outcome: age, sex group, procedure group, the broad composite outcome, log-transformed hospital length of stay and log-transformed anesthesia duration, each with a missingness indicator, patient class, and ICU admission. Stabilized weights were calculated as the marginal probability of complete-case inclusion divided by each encounter’s model-predicted probability. The primary outcome model was then refitted in the 5,349 complete cases using these weights and sandwich-robust standard errors. Weight distributions, effective sample size, and absolute standardized mean differences were examined, and truncation at the 1st and 99th percentiles was evaluated as an additional sensitivity analysis.

Data extraction, cleaning, and dataset preparation were performed using DuckDB and Python. Statistical analyses, including descriptive analyses, logistic regression models, sensitivity analyses, model diagnostics, and graphical outputs, were conducted in Python using pandas, NumPy, SciPy, statsmodels, and patsy. All tests were two-sided, and p values <0.05 were considered statistically significant.

### Ethics

This study was a secondary analysis of de-identified data from the Medical Informatics Operating Room Vitals and Events Repository (MOVER). The original MOVER data collection was approved by the Institutional Review Boards at the University of California, Irvine Medical Center and the main University of California, Irvine campus, and the requirement for individual patient consent was waived because protected health information was removed or de-identified in a HIPAA-compliant manner. The MOVER EPIC data were accessed for research purposes on 30 March 2026. The authors had access only to de-identified data and did not have access to information that could identify individual participants during or after data collection or analysis. The present study involved only secondary analysis of de-identified publicly available/controlled-access data and did not involve direct patient contact, new data collection, or access to identifiable private information. Therefore, additional institutional review board approval and informed consent were not required for this secondary analysis. All analyses were conducted in accordance with the MOVER data-use requirements.

## Results

### Cohort derivation and final analytic sample

Following abdominal surgery screening, exclusion of encounters involving individuals younger than 18 years, and case-level deduplication, the final adult analytic cohort included 5679 surgical encounters. This cohort was used for descriptive analyses and outcome frequency summaries. For the primary multivariable analysis, standardized ASA classification and complete-case restriction yielded 5,349 encounters. A larger complete-case cohort of 5,365 encounters was used for the ASA-excluded sensitivity analysis.

### Frequency of EHR-captured postoperative adverse-event indicators

In the deduplicated adult analytic cohort of 5679 surgical encounters, the broad EHR-captured postoperative adverse-event indicator composite was present in 166 encounters (2.92%). The narrow severe EHR-captured adverse-event indicator composite was present in 95 encounters (1.67%), and death was recorded in 23 encounters (0.40%). These outcome frequencies are summarized in Table 1.

**Table 1:** Frequency of EHR-recorded postoperative adverse-event indicators in the deduplicated adult analytic cohort.

Outcome	Events, n	Frequency, %
Broad EHR-captured postoperative adverse-event indicator composite	166	2.92
Narrow severe EHR-captured adverse-event indicator composite	95	1.67
Death recorded in the postoperative adverse-event data	23	0.40

**Note:** Outcomes were summarized in the deduplicated adult analytic cohort (n = 5679). Frequencies represent EHR-recorded complication indicators captured within the indexed perioperative encounter and should not be interpreted as standardized 30-day postoperative complication rates. Death was included in the composite outcomes when recorded and was also reported separately as a secondary descriptive outcome. Each composite outcome was coded at the case level.

These frequencies reflect complication indicators captured within the indexed perioperative encounter and should not be interpreted as standardized 30-day postoperative complication rates. Component-level distributions of the broad and narrow EHR-captured postoperative adverse-event indicator composites are provided in Supplementary Table S4b in S1 File.

### Baseline characteristics according to broad EHR-captured adverse-event indicator status

Baseline and perioperative characteristics stratified by broad EHR-captured postoperative adverse-event indicator status are summarized in Table 2. Compared with encounters without broad EHR-captured adverse-event indicators, encounters with broad EHR-captured adverse-event indicators were more frequently male, more often classified as ASA IV + , and showed a significantly different distribution across procedure groups. Encounters with complications also had a substantially longer hospital length of stay. In contrast, age, operating-room duration, and anesthesia duration did not differ significantly between groups.

**Table 2:** Perioperative characteristics and postoperative length of stay according to broad EHR-captured adverse-event indicator status.

Variable	Overall	No broad EHR-captured adverse-event indicator	Broad EHR-captured adverse-event indicator	P value
Sex, n (%)				0.001
Female	2850 (50.19)	2788 (50.58)	62 (37.35)	
Male	2828 (49.81)	2724 (49.42)	104 (62.65)	
ASA group, n (%)				<0.001
ASA I–II	2193 (40.70)	2174 (41.50)	19 (12.67)	
ASA III	2431 (45.12)	2382 (45.48)	49 (32.67)	
ASA IV+	764 (14.18)	682 (13.02)	82 (54.67)	
Procedure group, n (%)				<0.001
Low-complexity common abdominal procedures	2558 (45.04)	2527 (45.84)	31 (18.67)	
Intestinal resection/stoma procedures	1382 (24.34)	1356 (24.60)	26 (15.66)	
Laparotomy/adhesiolysis	1304 (22.96)	1208 (21.91)	96 (57.83)	
Upper abdominal major surgery	435 (7.66)	422 (7.65)	13 (7.83)	
Length of stay, median (IQR), days	3.00 (1.00–8.00)	3.00 (1.00–7.00)	8.00 (1.00–26.00)	<0.001
Operating-room duration, median (IQR), h	3.00 (2.13–4.43)	3.00 (2.13–4.42)	3.28 (2.37–4.84)	0.119
Anesthesia duration, median (IQR), h	3.13 (2.27–4.57)	3.13 (2.27–4.57)	2.98 (2.10–4.35)	0.132
Age, median (IQR), years	54.00 (38.00–65.00)	54.00 (38.00–66.00)	54.00 (39.00–65.00)	0.255

Note: Continuous variables are presented as median (IQR), and categorical variables are presented as n (%). Percentages for categorical variables were calculated within each column among records with non-missing data for that variable. The primary stratification variable was the broad EHR-captured postoperative adverse-event indicator composite. One record coded as “Other” for sex was excluded from sex-specific comparative analyses. Chi-square tests were used for categorical variables, and Mann–Whitney U tests were used for continuous variables. Variable-specific analyses were based on available-case data.

### Variable completeness and modeling dataset

Model-specific sample sizes and event counts differed because of the complete-case requirements applied to each analysis. The primary complete-case cohort included 5,349 encounters and 147 broad EHR-captured adverse-event indicator events, whereas the larger ASA-excluded sensitivity cohort included 5,365 encounters and 148 broad events. The secondary narrow-outcome model was fitted in the same primary complete-case cohort and included 84 narrow severe adverse-event indicator events. Full model-specific denominators, event counts, and event rates are summarized in Supplementary Table S8b in S1 File.

### Univariable associations with the broad EHR-captured adverse-event indicator composite

Univariable logistic regression analyses were conducted in the primary complete-case cohort to assess crude associations with the primary outcome, defined as the broad EHR-captured postoperative adverse-event indicator composite. Male sex was associated with higher odds of the captured broad composite in crude analysis. ASA group showed the strongest univariable association: compared with ASA III, ASA IV + was associated with substantially increased odds of EHR-captured adverse-event indicators, whereas ASA I–II was associated with lower odds. The procedure group also showed crude associations with the primary outcome, with higher odds observed for laparotomy/adhesiolysis and upper abdominal major surgery compared with low-complexity common abdominal procedures. Age and operating-room duration were not significantly associated with the primary outcome in univariable analysis. Full univariable results are presented in Supplementary Table S10 in S1 File.

### Multivariable logistic regression for the primary outcome

A primary multivariable logistic regression model adjusted for age, sex group, ASA group, procedure group, and operating-room duration was fitted in the primary complete-case cohort (n = 5,349) to evaluate independent associations with the broad EHR-captured postoperative adverse-event indicator composite.

After adjustment for the other covariates, ASA IV+ remained the strongest independent correlate of the primary outcome. Compared with ASA III, ASA IV + was associated with higher odds of the broad composite outcome (adjusted OR 4.66, 95% CI 2.92–7.45; P < 0.001), whereas ASA I–II was associated with lower odds (OR 0.55, 95% CI 0.31–0.99; p = 0.047). Laparotomy/adhesiolysis was also associated with higher odds compared with low-complexity common abdominal procedures (OR 1.79, 95% CI 1.02–3.13; p = 0.041). Age, sex, operating-room duration, intestinal resection/stoma procedures, and upper abdominal major surgery were not independently associated with the primary outcome in the conventional multivariable model. Detailed results are presented in Table 3.

**Table 3:** Multivariable logistic regression results for the primary outcome.

Variable	Adjusted OR	95% CI	P value
Age, per year	1.002	0.99–1.01	0.764
Operating-room duration, per hour	1.029	0.96–1.11	0.439
Male vs Female	1.136	0.80–1.62	0.481
Intestinal resection/stoma	0.899	0.49–1.64	0.727
Laparotomy/adhesiolysis	1.789	1.02–3.13	0.041
Upper abdominal major surgery	1.389	0.61–3.14	0.430
ASA IV + vs ASA III	4.661	2.92–7.45	<0.001
ASA I–II vs ASA III	0.550	0.31–0.99	0.047

Note: The dependent variable was the broad EHR-captured postoperative adverse-event indicator composite. The model was fitted in the primary complete-case adult cohort (n = 5,349) and included age, sex group, ASA group, procedure group, and operating-room duration. Age and operating-room duration were modeled as continuous variables. Female sex, ASA III, and low-complexity common abdominal procedures served as reference categories. Odds ratios and 95% confidence intervals were obtained by exponentiating the logistic regression coefficients. Full univariable results are provided in Supplementary Table S10 in S1 File.

Given the moderate number of outcome events, additional assessments were performed to evaluate model stability. The primary logistic regression model included 147 outcome events and 8 model parameters, corresponding to an events-per-parameter ratio of 18.4. No convergence issues or evidence of complete or quasi-complete separation were identified. Multicollinearity was not observed, with all variance inflation factors below 2.

In the inverse-probability-weighted analysis, the target cohort comprised 5,678 encounters with Female or Male sex coding, of which 5,349 met the primary complete-case definition. The stabilized weights were well behaved (mean 1.000, SD 0.035; range 0.949–1.178), no weight exceeded 1.25, and the effective sample size was 5,342.6. The maximum absolute standardized mean difference across variables included in the selection model decreased from 0.024 before weighting to 0.001 after weighting. The weighted estimates were similar to the primary analysis: ASA IV+ remained strongly associated with the broad composite compared with ASA III (OR 4.78, 95% CI 3.06–7.48; p < 0.001), and laparotomy/adhesiolysis remained associated with higher odds compared with low-complexity common abdominal procedures (OR 1.81, 95% CI 1.02–3.19; p = 0.042). The point estimate for ASA I–II was nearly unchanged, although its robust confidence interval included 1 (OR 0.55, 95% CI 0.29–1.03; p = 0.061). Truncation of weights at the 1st and 99th percentiles produced materially similar estimates (Supplementary Table S19 in S1 File).

### Sensitivity analysis

A sensitivity analysis excluding ASA from the multivariable model was conducted to assess the influence of ASA-related missingness and model specification. In the larger ASA-excluded sensitivity-analysis cohort (n = 5,365; broad composite events = 148), laparotomy/adhesiolysis was associated with substantially higher odds of broad composite compared with low-complexity common abdominal procedures (OR 5.86, 95% CI 3.80–9.04; p < 0.001). Male sex showed a modest association (OR 1.43, 95% CI 1.01–2.02; p = 0.042), while upper abdominal major surgery showed a borderline association (OR 2.20, 95% CI 1.00–4.82; p = 0.050). Age, operating-room duration, and intestinal resection/stoma procedures were not independently associated with the outcome (Supplementary Table S11 in S1 File).

In the restricted-sample ASA-excluded model fitted within the primary complete-case cohort (n = 5,349; broad composite events = 147), the overall direction of associations was broadly similar. Laparotomy/adhesiolysis remained strongly associated with the broad composite (OR 6.09, 95% CI 3.93–9.44; p < 0.001), and upper abdominal major surgery was associated with higher odds (OR 2.28, 95% CI 1.03–5.01; p = 0.041). The association for male sex was borderline (OR 1.41, 95% CI 1.00–1.99; p = 0.051), whereas age, operating-room duration, and intestinal resection/stoma procedures remained non-significant. Because the larger and same-sample ASA-excluded models produced similar estimates despite only a small difference in analytic sample size, the contrast with the primary ASA-adjusted model appears to reflect predominantly the influence of ASA adjustment rather than sample-size differences (Supplementary Table S12 in S1 File).

Centered restricted cubic spline analyses provided modest evidence of departure from linearity for age. Compared with the linear specification, the age spline model showed improved fit (likelihood-ratio χ² = 6.39, df = 2; p = 0.041), whereas the corresponding evidence for operating-room duration was weaker and did not reach statistical significance (χ² = 4.91, df = 2; p = 0.086). A model in which both variables were represented using centered spline terms also improved fit compared with the model containing both linear terms (χ² = 11.11, df = 4; p = 0.025). Nevertheless, the principal adjusted estimates remained materially unchanged. In the age-spline model, the ORs were 4.79 (95% CI 3.00–7.67) for ASA IV + , 0.55 (95% CI 0.30–0.99) for ASA I–II, and 1.79 (95% CI 1.03–3.14) for laparotomy/adhesiolysis. The corresponding estimates in the joint spline model were 4.80 (95% CI 3.01–7.66), 0.56 (95% CI 0.31–1.00), and 1.75 (95% CI 1.00–3.06), respectively.

Results were also robust to analyses addressing repeated encounters within patients. Patient-level cluster-robust standard errors produced effect estimates identical to the primary model and generally similar inference; ASA IV+ remained strongly associated with the broad composite, whereas the laparotomy/adhesiolysis association became borderline (OR 1.79, 95% CI 0.99–3.23; p = 0.053). In the first-encounter-per-patient sensitivity analysis (n = 4,704; broad composite events = 108), ASA IV+ remained strongly associated with the outcome (OR 4.67, 95% CI 2.72–8.01), and the overall pattern of estimates remained consistent with the primary analysis (Supplementary Tables S17 and S19 in S1 File).

### Secondary analysis using the narrow severe EHR-captured adverse-event indicator composite

A secondary exploratory analysis was conducted using the narrow severe EHR-captured adverse-event indicator composite as the dependent variable. The multivariable model used the same covariate structure as the primary model and was fitted in the primary complete-case cohort (n = 5,349; narrow severe adverse-event indicator events = 84).

In the adjusted model, clinician-assigned ASA physical status remained the strongest correlate of the narrow outcome. Compared with ASA III, ASA IV + was associated with higher odds of the narrow composite (OR 4.87, 95% CI 2.63–9.03; p < 0.001), whereas ASA I–II was associated with lower odds (OR 0.29, 95% CI 0.10–0.90; p = 0.032). Male sex was associated with higher odds compared with female sex (OR 1.83, 95% CI 1.09–3.05; p = 0.022), and laparotomy/adhesiolysis was associated with higher odds compared with low-complexity common abdominal procedures (OR 3.42, 95% CI 1.43–8.20; p = 0.006). Age, operating-room duration, intestinal resection/stoma procedures, and upper abdominal major surgery were not independently associated with the narrow outcome.

Because the narrow model contained fewer outcome events than the primary broad-composite model, these findings were considered exploratory and supportive rather than definitive. Full univariable and multivariable results are presented in Supplementary Tables S13 and S14 in S1 File.

## Discussion

### Principal findings

In this retrospective cohort study of adult abdominal surgery encounters from the EPIC component of MOVER, broad EHR-recorded postoperative adverse-event indicators were captured in 2.92% of the deduplicated adult analytic cohort. In the primary multivariable model, clinician-assigned ASA IV + was the most consistent correlate of the broad composite. Laparotomy/adhesiolysis was also associated with higher odds in the conventional model, although this weaker procedure-related association became borderline under patient-level cluster-robust inference and in some alternative model specifications. Chronological age, sex, operating-room duration, intestinal resection/stoma procedures, and upper abdominal major surgery were not independently associated with the broad composite in the primary adjusted model. ASA-excluded analyses produced substantially stronger procedure-group associations, particularly for laparotomy/adhesiolysis, suggesting that part of the crude operative-context signal overlapped with information reflected in clinician-assigned ASA physical status. This pattern suggests that some demographic and operative-context signals overlapped with information reflected in clinician-assigned ASA physical status, including comorbidity burden and perceived perioperative vulnerability. In the secondary analysis using the narrow severe EHR-captured adverse-event indicator composite, ASA remained strongly associated with the outcome, while male sex and laparotomy or adhesiolysis also showed supportive associations in the exploratory secondary model. Overall, these findings suggest that captured perioperative morbidity signals in this heterogeneous adult abdominal surgery cohort were more closely associated with clinician-assigned ASA physical status and operative context than with chronological age or case duration alone.

### ASA, age, and perioperative risk context

The prominence of ASA in the primary model is consistent with previous evidence showing that ASA physical status is associated with postoperative morbidity and mortality across surgical populations. Earlier perioperative studies have reported graded relationships between higher ASA class and adverse postoperative outcomes, and large database analyses have shown that ASA may retain prognostic information after adjustment for procedure-related characteristics [9–11]. In the present study, clinician-assigned ASA IV+ remained strongly associated with the broad EHR adverse-event composite after adjustment for chronological age and other prespecified covariates, whereas age itself was not independently associated with the primary outcome. This finding should not be interpreted as evidence that ASA directly measures physiological reserve or frailty. Rather, ASA is a pragmatic and partly subjective clinical classification that may reflect several overlapping dimensions, including comorbidity burden, clinician judgment, functional status, and perceived perioperative vulnerability. This interpretation is compatible with broader surgical literature showing that frailty and reduced physiological reserve are associated with postoperative morbidity independently of chronological age [12,13], as well as recent EHR-based evidence linking frailty-related measures with postsurgical adverse events [14]. Accordingly, ASA in this study is best viewed as a clinician-assigned summary of perioperative risk context rather than a direct measure of frailty or baseline physiological vulnerability [15].

### Procedure group, operative context, and ASA attenuation

The attenuation of procedure-group associations after adjustment for ASA should not be interpreted as evidence that operative context is clinically irrelevant. In the univariable analysis, laparotomy/adhesiolysis and upper abdominal major surgery were associated with higher odds of broad EHR-captured adverse-event indicators, and laparotomy/adhesiolysis remained associated with higher odds in the ASA-excluded sensitivity models. This pattern suggests that part of the crude procedure-related signal overlapped with information reflected in clinician-assigned ASA physical status, including comorbidity burden and perceived perioperative vulnerability. Such an interpretation is consistent with the E-PASS framework, which conceptualizes postoperative morbidity as the result of an imbalance between physiological reserve and surgical stress [5], and with evidence from HPB surgery showing that preoperative risk and surgical stress components are associated with severe postoperative complications [16]. It is also supported by evidence that frailty and operative stress jointly shape postoperative outcomes, rather than acting as isolated risk domains [17]. In this context, procedure group may be best understood as a broad marker of operative context whose apparent association with postoperative adverse-event indicators is partly shaped by underlying case mix and clinician-assessed perioperative risk.

### Operating-room duration and length of stay

The absence of a stable association between operating-room duration and postoperative complications warrants consideration. Operating-room duration was retained as a prespecified, clinically interpretable proxy for procedural exposure and operative complexity; however, it was not independently associated with either the broad composite outcome or the narrow severe-complication outcome. This finding argues against a simple “longer operation, higher risk” interpretation in this heterogeneous abdominal surgery cohort. A more plausible explanation is that case duration alone is an incomplete marker of surgical stress, which may also depend on procedural invasiveness, urgency, blood loss, tissue trauma, hemodynamic instability, infection burden, and baseline physiological reserve. In contrast, hospital length of stay was substantially longer among encounters with postoperative complications. This observation is clinically plausible and consistent with large-scale surgical registry evidence showing that postoperative complications are associated with increased risk-adjusted length of stay [18]. However, length of stay was not included as a predictor in the regression models because it is better interpreted as a postoperative consequence and marker of clinical burden rather than as a baseline or intraoperative risk factor.

### Narrow severe-complication outcome and clinical implications

The secondary analysis using the narrow severe EHR-captured adverse-event indicator composite provides additional clinical context. Surgical complication research has long emphasized that postoperative adverse events should not be interpreted solely as present or absent, but also in terms of their clinical severity and consequences [19]. In the present study, when the outcome definition was restricted to more severe postoperative events, ASA remained strongly associated with the narrow severe-complication outcome, while male sex and laparotomy/adhesiolysis also showed supportive associations in the exploratory secondary model. This pattern suggests that demographic and operative-context signals may become more apparent when the outcome focuses on clinically more consequential events. However, because the number of narrow severe-complication events was smaller than that of the broad composite outcome, these findings should be interpreted as supportive rather than definitive. Clinically, the results do not support using these variables as a stand-alone prediction tool. Instead, they suggest that simple perioperative information, particularly ASA status interpreted alongside operative context, may help clinicians frame postoperative risk, guide perioperative vigilance, and support recognition of patients who may require closer early postoperative monitoring.

### Strengths, limitations, and future directions

This study has several strengths. It used a publicly available perioperative database, focused on clinically interpretable variables rather than high-dimensional prediction modeling, and examined postoperative complication signals across a heterogeneous adult abdominal surgery cohort. The inclusion of multivariable adjustment, ASA-excluded sensitivity analyses, and a narrower severe-complication outcome provided additional context for evaluating the robustness and interpretation of the primary findings. The study also explicitly accounted for procedural heterogeneity by grouping abdominal procedures into clinically coherent categories rather than treating the cohort as procedurally uniform.

Several limitations should also be acknowledged. First, this was a retrospective single-center study based on routinely collected electronic health record data, and residual confounding cannot be excluded. Therefore, the primary ASA-adjusted model should be interpreted as an internally consistent complete-case analysis rather than as an unbiased estimate for the entire abdominal surgery cohort. Second, the analysis was limited to the EPIC component of MOVER and did not incorporate high-frequency waveform data or more granular intraoperative physiological variables, which may better capture intraoperative stress than operating-room duration alone. Third, postoperative complications were derived from database indicators rather than prospectively adjudicated clinical events, and the dataset lacked a standardized 30-day postoperative ascertainment window. Therefore, the observed frequency of complication indicators should be interpreted as captured morbidity within the indexed perioperative encounter rather than as a complete 30-day postoperative complication rate. It should not be directly compared with complication rates from NSQIP, ISSOS, or other registry studies using standardized follow-up, because complication definitions, ascertainment windows, and retrieval methods differ across data sources. Prior studies have shown that postoperative complication reporting can differ between clinical registries and administrative claims data, and that definitions and retrieval methods can materially influence complication statistics [20,21].

Fourth, the primary analysis used complete cases and excluded 330 of 5,679 encounters (5.8%). Excluded encounters had a higher frequency of the broad composite, longer hospital stays, and a greater representation of laparotomy/adhesiolysis procedures than encounters included in the primary model. Complete-case selection may therefore have under-represented higher-risk encounters and could have affected procedure-related estimates if missingness was associated with both operative characteristics and postoperative morbidity. However, the excluded proportion was relatively small, and the stabilized inverse-probability weights showed good overlap, preserved nearly the full effective sample size, and reduced measured covariate imbalance. The weighted effect estimates for ASA IV+ and laparotomy/adhesiolysis were close to those from the primary model, suggesting that the main findings were not explained by observed differences between included and excluded encounters. Nevertheless, IPW cannot address selection related to unmeasured factors, and the confidence interval for ASA I–II included 1 in the weighted analysis. The inverse association for ASA I–II and other weaker or borderline associations should therefore be interpreted cautiously.

In addition, age and operating-room duration were specified as linear continuous covariates in the primary model.

Centered restricted cubic spline analyses suggested modest nonlinearity in the association between age and the broad composite, whereas evidence of nonlinearity for operating-room duration was weaker. Importantly, the principal ASA and procedure-group estimates remained materially unchanged under the age-spline and joint-spline specifications. Therefore, the originally specified linear model was retained as the primary parsimonious analysis, while the spline models were treated as sensitivity analyses. The association with age should nevertheless not be summarized solely by a single per-year odds ratio.

Future research should validate these findings in external perioperative datasets and in more granular procedure-specific cohorts. Studies using prospectively adjudicated or registry-defined postoperative outcomes would help clarify whether the observed associations persist under standardized 30-day complication definitions. Further work could also integrate baseline physiological status with more detailed intraoperative measures, including hemodynamic instability, blood loss, procedure urgency, and high-frequency physiologic data, to better characterize the interaction between patient vulnerability and operative stress.

## Conclusion

In this retrospective cohort of adult abdominal surgery encounters from the MOVER EPIC component, clinician-assigned ASA physical status was the most consistent correlate of EHR-recorded postoperative adverse-event indicators. ASA should be interpreted as a pragmatic clinical classification that may reflect comorbidity burden, clinician judgment, and perceived perioperative vulnerability rather than as a direct measure of physiological reserve. Procedure-group associations varied across adjusted and sensitivity analyses, indicating that operative context may provide additional information but that weaker procedure-related associations should be interpreted cautiously. Operating-room duration did not show a stable independent association across models. Overall, these findings support cautious interpretation of routinely available perioperative variables when using EHR-derived morbidity signals in heterogeneous abdominal surgery populations.

## Supporting information

S1 File Supplementary tables and reporting checklist. This Microsoft Word file contains Supplementary Tables S1–S20, including cohort-construction rules, outcome definitions, variable derivations, missing-data and model-specific cohort summaries, regression and sensitivity analyses, the STROBE checklist, model-stability diagnostics, and detailed procedure-group definitions.
(DOCX)

## Abbreviation:

ASA: American Society of Anesthesiologists
CI: Confidence interval
EPIC: Electronic medical record component of MOVER
EHR: Electronic health record
ICU: Intensive care unit
IQR: Interquartile range
LOG_ID: Surgical encounter identifier
MOVER: Medical Informatics Operating Room Vitals and Events Repository
OR: odds ratio
STROBE: Strengthening the Reporting of Observational Studies in Epidemiology
