# Supplementary material for: Routinely available perioperative variables and EHR-captured postoperative adverse-event indicators after adult abdominal surgery: A retrospective cohort study using MOVER EPIC
Source: PLoS One. 2026 Oct 21;21(10):e0350704. doi: 10.1371/journal.pone.0350704 (PMC13686507; doi:10.1371/journal.pone.0350704)
Supplement: Supplementary file 1 [file pone.0350704.s001.docx]

**Table S 1. Case-level deduplication and cohort construction rules**

| Step | Process | Operational rule | Main field(s) used | Notes |
| --- | --- | --- | --- | --- |
| 1 | Data source selection | The EPIC component of the MOVER dataset was used for analysis | Dataset source | EPIC records correspond to surgical encounters from 2017 to 2022 |
| 2 | Define case-level analytic unit | LOG_ID was treated as the identifier for a single surgical encounter | LOG_ID | Case-level surgical encounter identifier |
| 3 | Deduplicate raw records | Only one final record was retained for each duplicated LOG_ID | LOG_ID | Each LOG_ID contributed one analytic record |
| 4 | Prioritisation during deduplication | Records with more complete procedure name, surgery date, and operating-room timing were preferentially retained | PRIMARY_PROCEDURE_NM, SURGERY_DATE, IN_OR_DTTM | Intended to improve variable derivation and outcome linkage |
| 5 | Identify candidate abdominal surgery records | Procedure-name rules were applied to identify abdominal surgical encounters | PRIMARY_PROCEDURE_NM | Detailed rules shown in Supplementary Table S2 |
| 6 | Exclude non-target procedures | Clearly non-abdominal and clinically irrelevant procedure groups were excluded | PRIMARY_PROCEDURE_NM | Examples include endoscopic, urologic, gynecologic, thoracic, neurosurgical, cardiac, thyroid, and other non-target operations |
| 7 | Construct final analytic cohort | Only records meeting the final abdominal surgery whitelist were retained | LOG_ID plus procedure-name rules | Final abdominal surgery cohort used for descriptive and regression analyses |

**Table S 2 Procedure-name whitelist used to define the abdominal surgery cohort**

| **Procedure category** | **Representative keywords / procedure examples** | **Procedure bucket used in analysis** | **Notes** |
| --- | --- | --- | --- |
| Cholecystectomy | cholecyst% | cholecystectomy | Includes gallbladder resection procedures |
| Appendectomy | appendect%, appendectomy% | appendectomy | Includes open or laparoscopic appendectomy |
| Colorectal resection | colect%, hemicolect%, sigmoidect%, cecect%, proctect%, low anterior resection, abdominoperineal resection | colorectal_resection | Includes major colon and rectal resections |
| Small bowel resection | bowel resection, small bowel resection, enterect% | small_bowel_resection | Includes small bowel resection procedures |
| Stoma-related surgery | ileostom%, colostom% | stoma_surgery | Includes stoma creation or closure |
| Laparotomy / adhesiolysis | laparotomy, exploratory laparotomy, lysis of adhesions | laparotomy_adhesiolysis | Often reflects emergency or complex abdominal surgery |
| Hernia repair | hernia% | hernia_repair | Restricted to abdominal-wall/abdominal hernia procedures |
| Gastrectomy | gastrect% | gastrectomy | Includes partial or total gastrectomy |
| Pancreatic surgery | pancreatect%, whipple% | pancreatic_surgery | Includes pancreatic resections and Whipple procedures |
| Splenectomy | splenect% | splenectomy | Includes splenic resection procedures |
| Endoscopic procedures | colonoscopy, egd, endoscop%, ercp | Not included | Diagnostic or endoscopic therapeutic procedures were excluded |
| Diagnostic laparoscopy and local examination procedures | laparoscopy, diagnostic, diagnostic laparoscopy, exam under anesthesia, anorectal | Not included | Excluded to improve cohort consistency |
| Foregut / bariatric procedures outside the target cohort | hiatal, paraesophageal, fundoplication, nissen, sleeve, bariatric, roux | Not included | Not retained in the final abdominal surgery whitelist |
| Urologic procedures | cystosc%, transurethral, turp, bladder, prostate, ureter, nephrect% | Not included | Outside target abdominal surgery scope |
| Gynaecologic procedures | salpingo%, oophor%, hystere%, uterus | Not included | Excluded as non-target pelvic/gynaecologic operations |
| Head and neck / endocrine procedures | thyroid%, parathyroid%, tonsil% | Not included | Outside abdominal surgical field |
| Thoracic procedures | lung, lobectomy, thorac%, vats | Not included | Excluded as thoracic surgery |
| Cardiac / cardiovascular procedures | atrial appendage, cath%, cabg, coronary, valve, pacemaker | Not included | Excluded as cardiac or cardiovascular procedures |
| Neurosurgical procedures | crani%, laminect%, spine, brain | Not included | Outside target surgical field |
| Other clearly non-target procedures | ophthalm%, cataract, mastect%, lumpect% | Not included | Excluded as non-abdominal procedures |

**Table S 3 Definitions and analytical roles of study outcomes**

| **Outcome type** | **Variable name** | **Outcome label** | **Operational definition** | **Analytical role** | **Notes** |
| --- | --- | --- | --- | --- | --- |
| Primary outcome | comp_system_broad | Broad EHR-captured adverse-event indicator composite | Case-level binary indicator identifying whether at least one database-recorded database-recorded postoperative adverse-event category was present, including cardiovascular, respiratory, airway, neurological, metabolic, injury/infection, or death-related events | Primary outcome | Intended as a broad measure of captured postoperative morbidity within the indexed perioperative encounter; not intended as a severity-graded endpoint |
| Secondary outcome | comp_severe_narrow | Narrow severe EHR-captured adverse-event indicator composite | Case-level binary indicator identifying whether at least one more clinically consequential postoperative complication item was present, including hemodynamic instability, hypotension, arrhythmia, cardiac arrest, hypoxemia, aspiration, hypercapnia, unplanned postoperative ventilation, reintubation, unplanned ICU admission recorded as a complication item, or death | Secondary / sensitivity-oriented outcome | Intended to provide a more restrictive outcome focused on severe or escalation-related database-recorded events |
| Secondary outcome | death_flag | Death | Binary indicator identifying cases explicitly marked as death in the postoperative complication record | Secondary descriptive outcome | Death was included in the composite outcomes when present and was also reported separately for transparency |
| Component of narrow composite / exploratory descriptor | Unplanned_icu-complication | Unplanned ICU admission complication | Binary indicator identifying unplanned ICU admission recorded in the postoperative complication data | Component of the Narrow severe EHR-captured adverse-event indicator composite | Treated as a severe component only when recorded as a postoperative complication item |
| Descriptive / supplementary variable | ICU_ADMIN_FLAG / icu_admin_true | ICU administrative indicator | Binary ICU-related administrative variable derived from EHR fields | Descriptive / supplementary variable | Not treated as a primary or secondary outcome because it may reflect planned ICU use, monitoring practice, or resource utilization rather than an adjudicated complication |

**Table S 4a. Components of the composite postoperative outcomes**

| Composite outcome | Component category / event | Clinical interpretation | Notes |
| --- | --- | --- | --- |
| comp_system_broad | Cardiovascular | Postoperative cardiovascular complication | Included in the Broad composite to capture cardiovascular morbidity recorded at the system-category level. Events within this category may differ in clinical severity; therefore, the category was used as a broad morbidity signal rather than as a uniformly severe endpoint. |
| comp_system_broad | Respiratory | Postoperative respiratory complication | Included to capture the broader range of database-recorded postoperative respiratory morbidity. More specific severe respiratory deterioration or escalation events were retained separately in the narrow composite. |
| comp_system_broad | Airway | Postoperative airway complication | Included because database-recorded airway events represent clinically relevant postoperative morbidity signals. The category was retained in the broad composite without assuming uniform severity across individual airway events. |
| comp_system_broad | Death | Death recorded in the postoperative complication data | Included because death represents an unambiguous severe postoperative outcome. Death was included in both composites and was also reported separately for transparency. |
| comp_system_broad | Neurological | Postoperative neurological complication | Included to capture neurological morbidity recorded in the postoperative adverse-event data. Because the category may contain events of differing severity, it was used as a broad morbidity indicator rather than a severe-event definition. |
| comp_system_broad | Metabolic | Postoperative metabolic complication | Included in the broad composite to capture database-recorded metabolic morbidity. It was not included as a category in the narrow composite because the source category did not consistently indicate severe postoperative deterioration. |
| comp_system_broad | Injury/Infection | Postoperative injury- or infection-related complication | Included to capture injury- and infection-related morbidity recorded in the postoperative adverse-event data. The higher-level category may encompass clinically heterogeneous events and therefore was retained only as part of the broad morbidity definition. |
| comp_severe_narrow | Instability (hemodynamic) | Haemodynamic instability | Included because acute hemodynamic instability represents clinically important physiological deterioration and is more specific for serious postoperative morbidity than a higher-level system category. |
| comp_severe_narrow | Hypotension | Clinically relevant postoperative hypotension | Included because postoperative hypotension represents a specific hemodynamic adverse-event indicator that may reflect clinically important circulatory compromise. |
| comp_severe_narrow | Arrhythmia | Postoperative cardiac rhythm disturbance | Included because a recorded postoperative arrhythmia represents a specific cardiovascular adverse event with potential clinical consequences and greater specificity than a broad cardiovascular category. |
| comp_severe_narrow | Arrest (cardiac) | Cardiac arrest | Included because cardiac arrest is an unambiguous, life-threatening postoperative cardiovascular event. |
| comp_severe_narrow | Hypoxemia | Postoperative hypoxaemia | Included because hypoxemia represents specific postoperative respiratory compromise rather than a nonspecific respiratory category. |
| comp_severe_narrow | Aspiration | Aspiration event | Included because aspiration represents a specific respiratory/airway adverse event with potential for substantial postoperative clinical deterioration. |
| comp_severe_narrow | Hypercapnia | Postoperative hypercapnia | Included because hypercapnia represents specific postoperative ventilatory compromise and provides greater clinical specificity than a broad respiratory category. |
| comp_severe_narrow | Unplanned postoperative ventilation | Unexpected postoperative ventilatory support | Included because unplanned postoperative ventilatory support indicates respiratory deterioration requiring escalation of care. |
| comp_severe_narrow | Reintubation / Reintubation (unplanned) | Unplanned postoperative airway re-establishment and ventilatory support | Included because reintubation indicates clinically important postoperative respiratory or airway deterioration requiring escalation of support. |
| comp_severe_narrow | Unplanned ICU admission | Unplanned ICU admission | Included because an ICU admission explicitly recorded as a postoperative adverse-event item represents unplanned escalation of care. Administrative ICU indicators were not used because they may represent planned ICU admission or routine monitoring. |
| comp_severe_narrow | Death | Death recorded in the complication data | Included in the narrow severe adverse-event indicator composite |

**Note:** Components represent postoperative adverse-event indicators recorded in the MOVER EPIC source data and should not be interpreted as externally adjudicated complications or standardized 30-day postoperative outcomes. The broad composite prioritized sensitivity to database-recorded morbidity signals, whereas the narrow composite prioritized more specific indicators of serious physiological deterioration or unplanned escalation of postoperative care. Individual encounters could contain more than one component event but contributed only once to each corresponding composite outcome. Unplanned ICU admission was included in the narrow composite only when recorded as a postoperative adverse-event item; administrative ICU indicators were not treated as outcomes.

**Supplementary Table S4b. Component-level frequencies of EHR-captured postoperative adverse-event indicators in the final adult analytic cohort**

| **Composite** | **Component** | **Encounters, n** | **% of final cohort** | **% among composite-positive encounters** |
| --- | --- | --- | --- | --- |
| Broad | Cardiovascular | 53 | 0.93 | 31.93 |
| Broad | Respiratory | 52 | 0.92 | 31.33 |
| Broad | Airway | 25 | 0.44 | 15.06 |
| Broad | Neurological | 7 | 0.12 | 4.22 |
| Broad | Metabolic | 6 | 0.11 | 3.61 |
| Broad | Injury/Infection | 26 | 0.46 | 15.66 |
| Broad | Death | 23 | 0.41 | 13.86 |
| Narrow | Hemodynamic instability | 30 | 0.53 | 31.58 |
| Narrow | Hypotension | 24 | 0.42 | 25.26 |
| Narrow | Arrhythmia | 10 | 0.18 | 10.53 |
| Narrow | Cardiac arrest | 11 | 0.19 | 11.58 |
| Narrow | Hypoxemia | 5 | 0.09 | 5.26 |
| Narrow | Aspiration | 12 | 0.21 | 12.63 |
| Narrow | Hypercapnia | 9 | 0.16 | 9.47 |
| Narrow | Unplanned postoperative ventilation | 10 | 0.18 | 10.53 |
| Narrow | Reintubation | 5 | 0.09 | 5.26 |
| Narrow | Unplanned ICU admission | 6 | 0.11 | 6.32 |
| Narrow | Death | 23 | 0.41 | 24.21 |

***Note:*** *Component frequencies were calculated in the final deduplicated adult analytic cohort (n = 5,679). Components were derived from database-recorded postoperative adverse-event fields and aligned with the prespecified broad and narrow EHR-captured adverse-event indicator composites. Encounters with multiple components could contribute to more than one component row; therefore, component counts are not mutually exclusive and may sum to more than the number of composite-positive encounters. Death was included in both the broad and narrow composites when recorded. The narrow composite included severe or escalation-related adverse-event indicators.*

**Table S 5 Candidate predictors and covariates: source fields, derivation, and analytical roles**

| Variable domain | Variable | Source field(s) | Processing / derivation | Analytical role | Notes |
| --- | --- | --- | --- | --- | --- |
| Demographic | Age | BIRTH_DATE, SURGERY_DATE | Calculated from surgery date and birth date; treated as a continuous variable when available | Descriptive analysis / candidate predictor | Age parsing and plausibility checks were performed during variable derivation |
| Demographic | Sex | SEX | Standardised into sex_group | Descriptive analysis / primary-model variable | Retained in the primary regression framework |
| Baseline clinical status | ASA physical status | ASA_RATING_C; ASA_RATING used for audit/label interpretation | Numeric ASA_RATING_C values were grouped as ASA I–II (codes 1–2), ASA III (code 3), and ASA IV+ (codes 4–6). | Descriptive analysis / primary-model variable | Numeric coding was used as the primary derivation source to avoid inconsistencies in text-based ASA descriptions. ASA was omitted in prespecified ASA-excluded sensitivity models. |
| Perioperative management | Primary anesthetic technique | PRIMARY_ANES_TYPE_NM | Collapsed where necessary into broader anesthesia categories | Descriptive analysis / supplementary covariate | Not prioritised in the parsimonious primary model |
| Perioperative management | Patient class | PATIENT_CLASS_GROUP, PATIENT_CLASS_NM | Collapsed where necessary into broader categories | Descriptive analysis / supplementary covariate | Not prioritised in the parsimonious primary model |
| Perioperative management | Operating-room duration | IN_OR_DTTM, OUT_OR_DTTM | Derived as OR duration and expressed in hours (or_hours) | Descriptive analysis / primary-model variable | Used as a proxy for procedural complexity and perioperative exposure burden |
| Perioperative management | Anesthesia duration | AN_START_DATETIME, AN_STOP_DATETIME | Derived as anesthesia duration and expressed in hours (an_hours) | Descriptive analysis / supplementary covariate | Considered relevant to perioperative exposure but not prioritised in the final parsimonious model |
| Procedure-related | Indexed procedure name | PRIMARY_PROCEDURE_NM | Used for abdominal surgery cohort construction and procedure identification | Cohort construction / descriptive analysis | Detailed inclusion and exclusion rules are shown in Supplementary Table S2 |
| Procedure-related | Procedure group | Derived from procedure-name whitelist rules and original procedure categories | Collapsed into a four-category modeling variable (procedure_group4) comprising low-complexity common abdominal procedures, intestinal resection/stoma procedures, upper abdominal major surgery, and laparotomy/adhesiolysis | Descriptive analysis / primary-model variable | Included to capture surgical heterogeneity while improving interpretability and reducing data sparsity; detailed mapping is provided in Supplementary Table S6 |
| Anthropometric | Height | HEIGHT | Extracted as recorded in the EHR | Descriptive analysis / exploratory variable | Not prioritised for the primary model because of higher missingness |
| Anthropometric | Weight | WEIGHT | Extracted as recorded in the EHR | Descriptive analysis / exploratory variable | Considered less problematic than height but not prioritised in the primary model |
| Anthropometric | Body mass index (if derived) | Derived from height and weight | Exploratory derivation only | Exploratory analysis | Use was limited by missingness, especially in height |
| ICU-related | ICU administrative indicator | ICU_ADMIN_FLAG | Recoded into a binary ICU-related variable (icu_admin_true) | Descriptive / supplementary analysis | Not treated as a primary outcome or core predictor |
| Hospitalisation-related | Length of stay | LOS | Treated as hospital length of stay as recorded in the EHR; converted to numeric format (los_num) | Descriptive analysis | More suitable as an outcome-related characteristic than as a primary baseline risk predictor |

**Table S 6 Derivation of the four-category procedure group used for modeling**

| original_procedure_bucket2 | procedure_group4 |
| --- | --- |
| appendectomy | low_complexity_common_abdominal |
| cholecystectomy | low_complexity_common_abdominal |
| hernia_repair | low_complexity_common_abdominal |
| colorectal_resection | intestinal_resection_stoma |
| small_bowel_resection | intestinal_resection_stoma |
| stoma_surgery | intestinal_resection_stoma |
| gastrectomy | upper_abdominal_major_surgery |
| pancreatic_surgery | upper_abdominal_major_surgery |
| Other (splenectomy) | upper_abdominal_major_surgery |
| laparotomy_adhesiolysis | laparotomy_adhesiolysis |

**Table S 7 Data cleaning and variable derivation rules**

| Processing domain | Variable / field | Processing rule | Derived output | Notes |
| --- | --- | --- | --- | --- |
| Timestamp parsing | “SURGERY DATE”, “BIRTH DATE” | Raw date fields were parsed and standardised | Basis for age derivation | Used to calculate age_years |
| Timestamp parsing | “IN OR DTTM”, “OUT OR DTTM” | Raw operating-room timestamps were parsed and standardised | Basis for OR duration derivation | Used to calculate or_duration_min |
| Timestamp parsing | “AN START DATETIME,”  “AN STOP DATETIME” | Raw anesthesia timestamps were parsed and standardised | Basis for anesthesia duration derivation | Used to calculate an_duration_min |
| Continuous-variable derivation | Age | Calculated from surgery date minus birth date and converted to years | Age years | Used as a continuous variable when available |
| Continuous-variable derivation | Operating-room duration | Calculated as operating-room exit time minus entry time | Or duration min, or hours | Used as a marker of operative complexity and perioperative exposure burden |
| Continuous-variable derivation | Anesthesia duration | Calculated as anesthesia stop time minus start time | An duration min,” an hours” | Used to characterise perioperative management exposure |
| Continuous-variable cleaning | Age | Implausible values were recoded as missing | Cleaned “age years” | Values outside the prespecified plausible range were set to missing |
| Continuous-variable cleaning | Operating-room duration | Implausible values were recoded as missing | Cleaned “or hours” | For example, non-positive or extreme values were set to missing |
| Continuous-variable cleaning | Anesthesia duration | Implausible values were recoded as missing | Cleaned an_hours | For example, non-positive or extreme values were set to missing |
| Continuous-variable cleaning | Length of stay | Converted to numeric format and checked for plausibility | los_num | Used mainly for descriptive analysis |
| Categorical standardisation | Sex | Raw sex values were mapped into standard analytical categories | sex_group | Typically grouped as Male, Female, or Other |
| Categorical recoding | ASA physical status | Numeric ASA_RATING_C values were used as the primary source for ASA classification. Codes 1–2 were assigned to ASA I–II, code 3 to ASA III, and codes 4–6 to ASA IV+. | asa_group_corrected | Numeric coding was preferred because text-based ASA descriptions showed inconsistent formatting. |
| Categorical collapsing | ASA grouped variable | Ordinal ASA categories were collapsed into broader clinical groups | ASA_I_II, ASA_III, ASA_IV_plus | Used to improve model stability and interpretability |
| Categorical recoding | ICU administrative indicator | Raw ICU-related field was converted into a binary indicator | icu_admin_true | Used for descriptive or supplementary analyses |
| Sparse-category handling | Procedure category | Infrequent categories were collapsed | procedure_bucket2 | Low-frequency levels were grouped as Other when needed |
| Sparse-category handling | Anesthetic technique | Infrequent categories were collapsed | primary_anes_group2 | Used mainly for descriptive or supplementary analyses |
| Sparse-category handling | Patient class | Infrequent categories were collapsed | patient_class_group2 | Used mainly for descriptive or supplementary analyses |
| Outcome standardisation | Comp system broad | Converted to a case-level binary variable | Broad EHR-captured adverse-event indicator composite | Primary outcome |
| Outcome standardisation | Comp severe narrow | Converted to a case-level binary variable | Severe postoperative complication composite | Secondary outcome |
| Outcome standardisation | Death flag | Converted to a case-level binary variable | Death | Secondary outcome |
| Outcome standardisation | Unplanned icu complication / icu admin true | Converted to case-level binary variables | ICU-related variables | Exploratory or descriptive use |

**Table S 8a. Missing data handling strategy and analytical implications**

| **Domain** | **Variable(s)** | **Available records, n (%)** | **Missing / analytically unavailable, n (%)** | **Missing-data approach** | **Analytical role** | **Notes / implications** |
| --- | --- | --- | --- | --- | --- | --- |
| Outcome variables | Broad composite; narrow severe composite; death | 5,679 (100%) | 0 (0%) | Outcomes were standardized as encounter-level binary indicators before analysis. | Primary and secondary outcomes | Outcome availability did not contribute to complete-case exclusion. |
| Demographic variable | Age | 5,679 (100%) | 0 (0%) | Age was analyzed as a continuous covariate in the primary model. | Primary-model covariate | No age-related complete-case exclusion. |
| Demographic variable | Sex group | 5,679 recorded | 1 (0.02%) not eligible for the male-versus-female contrast | Male and female categories were used in regression analyses; the single “Other” record was not included in the binary sex contrast. | Primary-model covariate | The “Other” record also had missing ASA and operating-room duration and therefore did not cause an additional complete-case exclusion. |
| Preoperative status | Corrected ASA group | 5,388 (94.9%) | 291 (5.1%) | Numeric ASA_RATING_C values were mapped as ASA I–II (1–2), ASA III (3), and ASA IV+ (4–6). Encounters without a resolvable ASA value were excluded from the primary complete-case model. | Primary-model covariate | ASA missingness overlapped substantially with operating-room-duration missingness. A larger ASA-excluded sensitivity analysis was performed. |
| Procedure variable | Four-group procedure classification | 5,679 (100%) | 0 (0%) | Procedure categories were assigned before regression modeling using the predefined four-group classification. | Primary-model covariate | Procedure-group availability did not contribute to complete-case exclusion. |
| Operative variable | Operating-room duration | 5,365 (94.5%) | 314 (5.5%) | Encounters without a valid operating-room duration were excluded from models requiring this covariate. | Primary-model covariate | Operating-room-duration missingness overlapped substantially with ASA missingness. |
| Missingness pattern | ASA and/or operating-room duration | — | 330 (5.8%) excluded overall | Complete-case analysis was used for the primary model. Among excluded encounters, 275 lacked both ASA and operating-room duration, 16 lacked ASA only, and 39 lacked operating-room duration only. | Primary complete-case cohort | The resulting primary cohort contained 5,349 encounters (94.2% of the adult analytic cohort). |
| Sensitivity cohort | Model excluding ASA from the complete-case requirement | 5,365 (94.5%) | 314 (5.5%) | ASA was omitted from the model and from the complete-case requirement. | Missing-data sensitivity analysis | This analysis retained encounters with missing ASA when all other required variables were available and was used to assess robustness to ASA-related exclusion. |

***Note:*** *Percentages use the deduplicated adult analytic cohort (n = 5,679) as the denominator unless otherwise specified. Missingness in ASA and operating-room duration overlapped substantially: 275 encounters lacked both variables, 16 lacked ASA only, and 39 lacked operating-room duration only, yielding 330 encounters excluded from the primary complete-case analysis and a final primary cohort of 5,349 encounters.*

**Supplementary Table S8b. Model-specific analytic cohorts and event counts**

| Analysis / model | Outcome | Analytic cohort definition | N | Events, n | Event rate, % | Purpose |
| --- | --- | --- | --- | --- | --- | --- |
| Full adult analytic cohort | Broad EHR-captured adverse-event indicator composite | Deduplicated adult abdominal surgery cohort | 5,679 | 166 | 2.92 | Descriptive outcome frequency |
| Full adult analytic cohort | Narrow severe EHR-captured adverse-event indicator composite | Deduplicated adult abdominal surgery cohort | 5,679 | 95 | 1.67 | Descriptive secondary outcome frequency |
| Primary complete-case model | Broad EHR-captured adverse-event indicator composite | Complete cases for age, sex group, ASA group, procedure group, and operating-room duration | 5,349 | 147 | 2.75 | Primary multivariable model |
| Primary univariable models | Broad EHR-captured adverse-event indicator composite | Same as primary complete-case cohort | 5,349 | 147 | 2.75 | Crude associations comparable with primary adjusted model |
| Restricted-sample ASA-excluded model | Broad EHR-captured adverse-event indicator composite | Same primary complete-case cohort, but ASA removed from model | 5,349 | 147 | 2.75 | Isolate effect of ASA adjustment while holding sample constant |
| Larger ASA-excluded sensitivity model | Broad EHR-captured adverse-event indicator composite | Complete cases for age, sex group, procedure group, and operating-room duration; ASA not required | 5,365 | 148 | 2.76 | Assess influence of ASA-related missingness |
| Categorized operating-room duration sensitivity model | Broad EHR-captured adverse-event indicator composite | Same as primary complete-case cohort | 5,349 | 147 | 2.75 | Test duration specification |
| First-encounter-per-patient sensitivity model | Broad EHR-captured adverse-event indicator composite | Earliest eligible encounter per patient from primary complete-case cohort | 4,704 | 108 | 2.30 | Assess influence of repeated encounters |
| Secondary narrow-outcome model | Narrow severe EHR-captured adverse-event indicator composite | Same as primary complete-case cohort | 5.349 | 84 | 1.57 | Supportive secondary analysis |

***Note:*** *Event rates were calculated within each model-specific analytic cohort. The broad and narrow outcomes were EHR-captured postoperative adverse-event indicator composites and should not be interpreted as standardized 30-day postoperative complication rates. The primary complete-case cohort required non-missing values for age, sex group, ASA group, procedure group, and operating-room duration. The larger ASA-excluded cohort did not require non-missing ASA.*

**Table S 9 Statistical analysis framework**

| Analysis component | Method | Variable type / target | Purpose | Notes |
| --- | --- | --- | --- | --- |
| Descriptive analysis | Summary statistics | Continuous variables | To describe the distribution of baseline and perioperative variables | Means, standard deviations, medians, and interquartile ranges were generated as appropriate |
| Descriptive analysis | Frequency tables | Categorical variables | To summarise baseline characteristics and outcome distributions | Reported as counts and percentages |
| Missingness assessment | Variable-level missingness summary | Prespecified analysis variables | To quantify data completeness before model construction | Performed after variable derivation and recoding |
| Outcome comparison | Stratified descriptive summaries | Primary outcome present vs absent | To compare cohort characteristics according to postoperative complication status | Continuous and categorical summaries were generated by outcome group |
| Univariable modeling | Logistic regression | Candidate predictors vs primary outcome | To estimate crude associations with the Broad EHR-captured adverse-event indicator composite | Results reported as ORs with 95% CIs and p values |
| Multivariable modeling | Logistic regression | Prespecified covariates vs primary outcome | To estimate adjusted associations with EHR-captured postoperative adverse-event indicators | Main model included age, sex group, ASA group, procedure group, and operating-room duration. |
| Outcome model | Binary logistic regression | comp_system_broad | Primary inferential analysis | Main outcome of interest |
| Supplementary model preparation | Recoding and broader model development | Additional perioperative and categorical variables | To assess whether broader covariate adjustment was feasible | Inclusion depended on completeness, distribution, and model stability |
| Variable preparation for modeling | Recoding / collapsing | Categorical predictors | To improve interpretability and model stability | Sparse levels were collapsed into broader categories such as Other where needed |
| Continuous-variable preparation | Unit conversion and plausibility filtering | Duration variables | To support interpretable modeling | Operating-room and anesthesia durations were expressed in hours |
| Missing-data strategy in modeling | Complete-case analysis | Selected regression variables | To define the final modeling dataset | No multiple imputation was performed |
| Software for data extraction and preparation | DuckDB and Python | Cohort construction, cleaning, and dataset preparation | To implement data extraction, deduplication, variable derivation, and analysis-ready dataset construction | Python was also used for intermediate model-development steps |
| Software for final statistical analyses | Python using pandas, NumPy, SciPy, statsmodels, and patsy | Descriptive analyses, logistic regression, sensitivity analyses, and model diagnostics | To generate the final results reported in the manuscript | Final tables and regression outputs were produced in python |
| Statistical significance | Two-sided hypothesis testing | All inferential analyses | To determine statistical evidence of association | Statistical significance defined as p < 0.05 |

**Table S 10 Full univariable logistic regression analyses for the primary outcome**

| **Variable** | **OR** | **95% CI** | **P value** |
| --- | --- | --- | --- |
| Age, per year | 1.007 | 0.995–1.019 | 0.279 |
| Male sex vs Female | 1.73 | 1.24–2.44 | 0.018 |
| ASA I–II vs ASA III | 0.45 | 0.26–0.77 | 0.004 |
| ASA IV+ vs ASA III | 6.27 | 4.33–9.09 | <0.001 |
| Intestinal resection/stoma procedures vs low-complexity common abdominal procedures | 1.31 | 0.73–2.23 | 0.36 |
| Laparotomy/adhesiolysis vs low-complexity common abdominal procedures | 6.41 | 4.18–9.84 | <0.001 |
| Upper abdominal major surgery vs low-complexity common abdominal procedures | 2.42 | 1.22–4.78 | 0.011 |
| Operating-room duration, per hour | 1.02 | 0.96–1.09 | 0.47 |

**Note:** The dependent variable was the Broad EHR-captured adverse-event indicator composite. Univariable logistic regression models were fitted in the same primary complete-case adult cohort used for the primary multivariable model (n = 5,349; broad composite events = 147). Age and operating-room duration were modeled as continuous variables. Female sex, ASA III, and low-complexity common abdominal procedures served as reference categories. Odds ratios and 95% confidence intervals were obtained by exponentiating the logistic regression coefficients.

**Table S 11 Sensitivity multivariable logistic regression for the primary outcome excluding ASA (n = 5365)**

| **Variable** | **Adjusted OR** | **95% CI** | **P value** |
| --- | --- | --- | --- |
| Age, per year | 1.01 | 1.00–1.02 | 0.157 |
| Operating-room duration, per hour | 0.99 | 0.92–1.06 | 0.708 |
| Male sex vs Female | 1.43 | 1.01–2.02 | 0.042 |
| Intestinal resection/stoma procedures vs low-complexity common abdominal procedures | 1.21 | 0.68–2.15 | 0.524 |
| Laparotomy/adhesiolysis vs low-complexity common abdominal procedures | 5.86 | 3.80–9.04 | <0.001 |
| Upper abdominal major surgery vs low-complexity common abdominal procedures | 2.20 | 1.00–4.82 | 0.050 |

**Note:** The dependent variable was the Broad EHR-captured adverse-event indicator composite. This sensitivity model excluded ASA group and was fitted in the larger complete-case adult cohort with non-missing age, sex group, procedure group, and operating-room duration(n = 5365; broad composite events = 148). Age and operating-room duration were modeled as continuous variables. Female sex and low-complexity common abdominal procedures served as reference categories. Odds ratios and 95% confidence intervals were obtained by exponentiating the logistic regression coefficients.

**Table S 12 ASA-excluded multivariable sensitivity analysis in the primary complete-case cohort**

| Variable | Adjusted OR | 95% CI | P value |
| --- | --- | --- | --- |
| Age, per year | 1.01 | 1.00–1.02 | 0.20 |
| Operating-room duration, per hour | 0.99 | 0.92–1.06 | 0.73 |
| Male sex vs Female | 1.41 | 1.00–1.99 | 0.05 |
| Intestinal resection/stoma procedures vs low-complexity common abdominal procedures | 1.25 | 0.70–2.23 | 0.46 |
| Laparotomy/adhesiolysis vs low-complexity common abdominal procedures | 6.09 | 3.93–9.44 | <0.001 |
| Upper abdominal major surgery vs low-complexity common abdominal procedures | 2.30 | 1.03–5.01 | 0.04 |

**Note:** ASA physical status was omitted from the model while retaining the same corrected primary complete-case cohort used for the main analysis (n = 5,349; broad composite events = 147). This analysis was used to distinguish the influence of ASA adjustment from differences in analytic sample size. Female sex and low-complexity common abdominal procedures served as reference categories..

**Table S 13 Univariable logistic regression analyses for the narrow severe-complication outcome**

| **Variable** | **OR** | | | **95% CI** | **P value** |
| --- | --- | --- | --- | --- | --- |
| Age, per year | 1.01 | 0.99–1.02 | | | 0.282 |
| Male sex vs Female | 3.12 | 1.90–5.13 | | | <0.001 |
| ASA I–II vs ASA III | 0.21 | 0.07–0.61 | | | 0.004 |
| ASA IV+ vs ASA III | 9.67 | 5.83–16.01 | | | <0.001 |
| Intestinal resection/stoma procedures vs low-complexity common abdominal procedures | 2.48 | | 0.996–6.19 | | 0.051 |
| Laparotomy/adhesiolysis vs low-complexity common abdominal procedures | 16.75 | | 7.99–35.11 | | <0.001 |
| Upper abdominal major surgery vs low-complexity common abdominal procedures | 2.16 | | 0.57–8.18 | | 0.26 |
| Operating-room duration, per hour | 0.996 | | 0.92–1.08 | | 0.93 |

**Note:** The dependent variable was the narrow severe postoperative complication composite. Univariable logistic regression models were fitted in the same primary complete-case adult cohort used for the narrow-outcome multivariable model (n = 5，349; narrow severe-complication events = 84). Age and operating-room duration were modeled as continuous variables. Female sex, ASA III, and low-complexity common abdominal procedures served as reference categories. Odds ratios and 95% confidence intervals were obtained by exponentiating the logistic regression coefficients.

**Table S 14 Multivariable logistic regression model for the narrow severe-complication outcome**

| Variable | Adjusted OR | 95% CI | P value |
| --- | --- | --- | --- |
| Age, per year | 1.005 | 0.993–1.018 | 0.412 |
| Operating-room duration, per hour | 1.027 | 0.929–1.134 | 0.606 |
| Male sex vs Female | **1.825** | **1.091–3.053** | **0.022** |
| Intestinal resection/stoma procedures vs low-complexity common abdominal procedures | 1.406 | 0.542–3.648 | 0.484 |
| Laparotomy/adhesiolysis vs low-complexity common abdominal procedures | **3.421** | **1.428–8.198** | **0.006** |
| Upper abdominal major surgery vs low-complexity common abdominal procedures | 0.940 | 0.218–4.043 | 0.933 |
| ASA I–II vs ASA III | **0.295** | **0.096–0.903** | **0.032** |
| ASA IV+ vs ASA III | **4.875** | **2.630–9.034** | **<0.001** |

**Note:** The dependent variable was the narrow severe postoperative complication composite. The model included age, sex group, ASA group, procedure group, and operating-room duration and was fitted in the primary complete-case adult cohort, n = 5,349 (narrow severe-complication events = 84). Age and operating-room duration were modeled as continuous variables. Female sex, ASA III, and low-complexity common abdominal procedures served as reference categories. Odds ratios and 95% confidence intervals were obtained by exponentiating the logistic regression coefficients.

**Table S 15 Comparison of encounters included in and excluded from the primary complete-case cohort**

| Variable | Included in primary complete-case cohort (n = 5,349) | Excluded from primary complete-case cohort (n = 330) | P value |
| --- | --- | --- | --- |
| Age, median (IQR), years | 54.0 (38.0–66.0) | 52.5 (37.0–63.0) | 0.247 |
| Sex, n (%) |  |  | **0.019** |
| Female | 2,706 (50.59) | 144 (43.77)* |  |
| Male | 2,643 (49.41) | 185 (56.23)* |  |
| Procedure group, n (%) |  |  | **<0.001** |
| Low-complexity common abdominal procedures | 2,412 (45.09) | 146 (44.24) |  |
| Intestinal resection/stoma procedures | 1,343 (25.11) | 39 (11.82) |  |
| Laparotomy/adhesiolysis | 1,174 (21.95) | 130 (39.39) |  |
| Upper abdominal major surgery | 420 (7.85) | 15 (4.55) |  |
| Broad EHR-captured adverse-event indicator composite, n (%) | 147 (2.75) | 19 (5.76) | **0.003** |
| Length of stay, median (IQR), days | 3.0 (1.0–7.0) | 5.0 (2.0–13.0) | **<0.001** |

**Note:** The primary complete-case cohort included encounters with non-missing values for the broad EHR-recorded postoperative complication indicator composite, age, sex group, ASA group, procedure group, and operating-room duration. The excluded group comprised encounters from the deduplicated adult analytic cohort that did not meet this complete-case definition. One encounter coded as “Other” for sex was excluded from the sex-specific comparison; therefore, female and male percentages in the excluded group were calculated using a denominator of 329, while the overall excluded cohort remained 330 encounters. Categorical variables are presented as n (%), with percentages calculated within each inclusion group. Continuous variables are presented as median (IQR). Chi-square tests were used for categorical variables, and Mann–Whitney U tests were used for continuous variables.

**Table S 16 Sensitivity analysis using categorized operating-room duration**

| Variable | Adjusted OR | 95% CI | P value |
| --- | --- | --- | --- |
| OR duration <2 h vs 2–4 h | 0.931 | 0.572–1.514 | 0.773 |
| OR duration >4 h vs 2–4 h | 1.331 | 0.897–1.976 | 0.156 |
| Age, per year | 1.002 | 0.992–1.012 | 0.754 |
| Male vs Female | 1.140 | 0.799–1.626 | 0.470 |
| Intestinal resection/stoma vs low-complexity | 0.840 | 0.458–1.541 | 0.573 |
| Laparotomy/adhesiolysis vs low-complexity | 1.718 | 0.982–3.004 | 0.058 |
| Upper abdominal major surgery vs low-complexity | 1.322 | 0.617–2.833 | 0.473 |
| ASA I–II vs ASA III | 0.561 | 0.312–1.010 | 0.054 |
| ASA IV+ vs ASA III | **4.791** | **3.008–7.632** | **<0.001** |

**Note:** The model was fitted in the corrected primary complete-case cohort (n = 5,349; broad composite events = 147). Operating-room duration was modeled categorically using 2–4 h as the reference category. Other covariates were identical to those in the primary multivariable model..

**Table S 17 First-encounter-per-patient sensitivity multivariable logistic regression for the primary outcome**

| Variable | Adjusted OR | 95% CI | P value |
| --- | --- | --- | --- |
| Age, per year | 1.009 | 0.997–1.021 | 0.125 |
| Operating-room duration, per hour | 1.041 | 0.960–1.128 | 0.336 |
| Male vs Female | 1.332 | 0.880–2.018 | 0.176 |
| Intestinal resection/stoma vs low-complexity | 0.683 | 0.336–1.390 | 0.293 |
| Laparotomy/adhesiolysis vs low-complexity | **1.897** | **1.022–3.520** | **0.042** |
| Upper abdominal major surgery vs low-complexity | 1.194 | 0.497–2.865 | 0.692 |
| ASA I–II vs ASA III | 0.583 | 0.304–1.118 | 0.105 |
| ASA IV+ vs ASA III | **4.670** | **2.722–8.011** | **<0.001** |

**Note:** The analysis was restricted to the earliest eligible encounter per patient from the corrected primary complete-case cohort (n = 4,704; broad composite events = 108). The model used the same covariate specification and reference categories as the primary multivariable analysis..

**Table S 18 STROBE checklist for cohort studies**

| Item No. | Recommendation | Page No. | Manuscript location |
| --- | --- | --- | --- |
| 1(a) | Indicate the study design with a commonly used term in the title or the abstract. | 1-2 | Title; Abstract, Methods |
| 1(b) | Provide in the abstract an informative and balanced summary of what was done and what was found. | 2 | Abstract |
| 2 | Explain the scientific background and rationale for the investigation being reported. | 3 | Introduction, paragraphs 1–2 |
| 3 | State specific objectives, including any prespecified hypotheses. | 4-5 | Introduction, final paragraph |
| 4 | Present key elements of study design early in the paper. | 6 | Methods 3.1 Study design and data source |
| 5 | Describe the setting, locations, and relevant dates, including periods of recruitment, exposure, follow-up, and data collection. | 6-8 | Methods 3.1 Study design and data source |
| 6(a) | Give the eligibility criteria, and the sources and methods of selection of participants. Describe methods of follow-up. | 6-8 | Methods 3.2 Cohort identification; Figure 1; Supplementary Tables S1–S2 |
| 6(b) | For matched studies, give matching criteria and number of exposed and unexposed. |  | Not applicable; this was not a matched cohort study |
| 7 | Clearly define all outcomes, exposures, predictors, potential confounders, and effect modifiers. Give diagnostic criteria, if applicable. | 8-12 | Methods 3.3 Outcome definitions; Methods 3.4 Candidate predictors and covariates; Supplementary Tables S3–S5 |
| 8 | For each variable of interest, give sources of data and details of methods of assessment or measurement. Describe comparability of assessment methods if there is more than one group. | 12-14 | Methods 3.1 Study design and data source; Methods 3.5 Data cleaning and variable derivation; Supplementary Tables S5–S7 |
| 9 | Describe any efforts to address potential sources of bias. | 14-18 | Methods 3.6 Missing data handling; Methods 3.7 Statistical analysis; Discussion, Strengths, limitations, and future directions; Supplementary Table S15 |
| 10 | Explain how the study size was arrived at. | 14-15 | Methods 3.2 Cohort identification; Methods 3.6 Missing data handling; Figure 1 |
| 11 | Explain how quantitative variables were handled in the analyses. If applicable, describe which groupings were chosen and why. | 11-12 | Methods 3.4 Candidate predictors and covariates; Methods 3.5 Data cleaning and variable derivation; Methods 3.7 Statistical analysis; Table 2; Supplementary Tables S5–S8 and S16a |
| 12(a) | Describe all statistical methods, including those used to control for confounding. | 16-18 | Methods 3.7 Statistical analysis; Table 3; Supplementary Tables S9–S17 |
| 12(b) | Describe any methods used to examine subgroups and interactions. |  | Not applicable; no subgroup or interaction analyses were performed |
| 12(c) | Explain how missing data were addressed. | 14 | Methods 3.6 Missing data handling; Results 4.4 Variable completeness and modeling dataset; Supplementary Tables S8 and S15 |
| 12(d) | If applicable, explain how loss to follow-up was addressed. | 24-26 | Not applicable; this was a retrospective EHR-based encounter-level analysis without prospective follow-up |
| 12(e) | Describe any sensitivity analyses. | 24-26 | Methods 3.7 Statistical analysis; Results 4.7 Sensitivity analysis; Supplementary Tables S11–S17 |
| 13(a) | Report numbers of individuals at each stage of study, including potentially eligible, examined for eligibility, confirmed eligible, included in the study, completing follow-up, and analysed. | 21-22 | Results 4.1 Cohort derivation and final analytic sample; Results 4.4 Variable completeness and modeling dataset; Figure 1 |
| 13(b) | Give reasons for non-participation at each stage. | 6-8 | Methods 3.2 Cohort identification; Figure 1; Supplementary Tables S1–S2 |
| 13(c) | Consider use of a flow diagram. | 8 | Figure 1 |
| 14(a) | Give characteristics of study participants and information on exposures and potential confounders. | 19 | Results 4.3 Baseline characteristics according to primary outcome status; Table 2; Supplementary Table S15 |
| 14(b) | Indicate number of participants with missing data for each variable of interest. | 19 | Methods 3.6 Missing data handling; Results 4.4 Variable completeness and modeling dataset; Supplementary Tables S8 and S15 |
| 14(c) | Summarise follow-up time, if applicable. |  | Not applicable; outcomes were interpreted as EHR-recorded complication indicators captured within the indexed perioperative encounter rather than standardized follow-up endpoints |
| 15 | Report numbers of outcome events or summary measures over time. | 19 | Results 4.2 Outcome incidence in the final cohort; Table 1; Results 4.4 for model-specific event counts |
| 16(a) | Give unadjusted estimates and, if applicable, confounder-adjusted estimates and their precision. Make clear which confounders were adjusted for and why. | 22-24 | Results 4.5 Univariable associations; Results 4.6 Multivariable logistic regression; Table 3; Supplementary Tables S10–S14 |
| 16(b) | Report category boundaries when continuous variables were categorized. | 24 | Methods 3.7 Statistical analysis; Supplementary Table S16 |
| 16(c) | If relevant, consider translating estimates of relative risk into absolute risk for a meaningful time period. |  | Not applicable; no standardized 30-day absolute risk estimate was calculated. Outcome frequencies are reported in Results 4.2 and Table 1 |
| 17 | Report other analyses done, such as subgroup analyses, interaction analyses, and sensitivity analyses. | 24-26 | Results 4.7 Sensitivity analysis; Results 4.8 Secondary analysis; Supplementary Tables S11–S17 |
| 18 | Summarise key results with reference to study objectives. | 27 | Discussion, Principal findings |
| 19 | Discuss limitations of the study, taking into account sources of potential bias or imprecision. | 31-33 | Discussion, Strengths, limitations, and future directions |
| 20 | Give a cautious overall interpretation of results considering objectives, limitations, multiplicity of analyses, results from similar studies, and other relevant evidence. | 33 | Discussion; Conclusion |
| 21 | Discuss the generalisability of the study results. | 31-33 | Discussion, Strengths, limitations, and future directions; Future research paragraph |
| 22 | Give the source of funding and the role of the funders for the present study and, if applicable, for the original study on which the present article is based. | 34-35 | Declarations, Funding |

Table S19 Model stability diagnostics and sensitivity analyses for the broad and narrow multivariable logistic regression models

Part A. Model diagnostic assessments

| **Assessment** | **Broad composite model** | **Narrow severe composite model** |
| --- | --- | --- |
| Analytic cohort | n = 5,349 | n = 5,349 |
| Outcome events | 147 | 84 |
| Modeled parameters, excluding intercept | 8 | 8 |
| Events per parameter | 18.4 | 10.5 |
| Maximum variance inflation factor | 1.84 | 1.84 |
| Minimum events in a categorical level | 12 | 3 |
| Minimum non-events in a categorical level | 408 | 417 |
| Zero-event or zero-non-event categorical cells | 0 | 0 |
| MLE convergence | Successful | Successful |
| Firth logistic regression convergence | Successful | Successful |
| Complete separation | Not detected | Not detected |
| Quasi-complete separation | Not detected | Not detected |

**Note:** Both models included age, sex group, ASA group, procedure group, and operating-room duration. The modeled parameter count excluded the intercept. No zero-event or zero-non-event categorical cells were identified, and neither complete nor quasi-complete separation was detected. Although the narrow-outcome model had fewer events and a minimum of three events in one categorical level, both maximum-likelihood and Firth penalized models converged successfully, with broadly similar principal estimates. The narrow-outcome analysis should nevertheless be interpreted as supportive because of its lower event count.

Part B. Sensitivity analysis summary

| **Analysis** | **Purpose** | **Main finding** |
| --- | --- | --- |
| Firth penalized logistic regression for the broad and narrow outcomes | Assess robustness to small-event bias, particularly for the narrow outcome | For both outcomes, the direction and magnitude of the principal estimates remained similar to the maximum-likelihood estimates. |
| Patient-level cluster-robust standard errors | Assess variance robustness in the presence of repeated encounters | The direction of associations was unchanged; the laparotomy/adhesiolysis estimate became borderline (OR 1.79, 95% CI 0.99–3.23; p = 0.053). |
| Stabilized inverse probability weighting with sandwich-robust standard errors | Assess sensitivity to complete-case selection conditional on measured variables | Weights were stable (range 0.949–1.178; effective sample size 5,342.6). Estimates for ASA IV+ and laparotomy/adhesiolysis were similar to the primary model; the confidence interval for ASA I–II included 1. |
| Exclusion of the two ASA VI encounters | Assess the influence of ASA VI coding on the ASA IV+ estimates | Results were materially unchanged: ASA IV+ OR 4.62 (95% CI 2.89–7.40) for the broad outcome and 4.81 (95% CI 2.59–8.94) for the narrow outcome. |

Part C1. Weight and balance diagnostics

| **Diagnostic** | **Result** |
| --- | --- |
| IPW target cohort | 5,678 encounters with Female or Male sex coding |
| Primary complete cases / non-complete cases | 5,349 / 329 |
| Marginal probability of complete-case inclusion | 0.9421 |
| Selection-model covariates | Age; sex group; procedure group; broad composite outcome; log-transformed hospital length of stay and log-transformed anesthesia duration, each with a missingness indicator; patient class; ICU admission |
| Predicted inclusion probability among complete cases | 0.800–0.992 |
| Stabilized weight, mean (SD) | 1.000 (0.035) |
| Stabilized weight, median (range) | 1.002 (0.949–1.178) |
| Weights >1.25 | 0 |
| Effective sample size | 5,342.6 of 5,349 |
| Maximum absolute SMD, before weighting | 0.024 |
| Maximum absolute SMD, after weighting | 0.001 |
| 1st/99th-percentile truncation bounds | 0.961 / 1.101 |
| Effective sample size after 1st/99th-percentile truncation | 5,343.1 |

Part C2. Primary and IPW-adjusted estimates for the broad composite outcome

| **Variable** | **Primary model OR (95% CI)** | **IPW model OR (95% CI)** | **IPW, 1st/99th truncated OR (95% CI)** |
| --- | --- | --- | --- |
| Age, per year | 1.002 (0.99–1.01) | 1.002 (0.99–1.01) | 1.001 (0.99–1.01) |
| Operating-room duration, per hour | 1.029 (0.96–1.11) | 1.031 (0.95–1.12) | 1.031 (0.95–1.12) |
| Male vs Female | 1.136 (0.80–1.62) | 1.136 (0.79–1.63) | 1.125 (0.78–1.61) |
| Intestinal resection/stoma vs low-complexity | 0.899 (0.49–1.64) | 0.882 (0.46–1.67) | 0.884 (0.47–1.68) |
| Laparotomy/adhesiolysis vs low-complexity | 1.789 (1.02–3.13) | 1.806 (1.02–3.19) | 1.789 (1.01–3.17) |
| Upper abdominal major surgery vs low-complexity | 1.389 (0.61–3.14) | 1.358 (0.55–3.37) | 1.364 (0.55–3.38) |
| ASA I–II vs ASA III | 0.550 (0.31–0.99) | 0.549 (0.29–1.03) | 0.548 (0.29–1.03) |
| ASA IV+ vs ASA III | 4.661 (2.92–7.45) | 4.781 (3.06–7.48) | 4.765 (3.05–7.46) |

**Note:** The primary model used conventional maximum-likelihood logistic regression. IPW models used stabilized weights and sandwich-robust standard errors. Female sex, ASA III, and low-complexity common abdominal procedures were the reference categories. The IPW target excluded the single encounter coded as “Other” for sex; this encounter also lacked ASA and operating-room duration and was already excluded from the primary model. SMD indicates standardized mean difference. Truncation was evaluated only as a secondary stability check because the untruncated weights showed no material extremes

Part D. Assessment of nonlinearity for continuous covariates

| **Continuous variable/model** | **Comparison** | **LR statistic** | **df** | **P value** |
| --- | --- | --- | --- | --- |
| Age | Linear vs centered restricted cubic spline | 6.39 | 2 | 0.041 |
| Operating-room duration | Linear vs centered restricted cubic spline | 4.91 | 2 | 0.086 |
| Age and operating-room duration | Both linear vs both centered spline specifications | 11.11 | 4 | 0.025 |

Part E. Stability of key non-spline estimates under alternative continuous-covariate specifications

| **Variable** | **Primary linear model OR (95% CI)** | **Age centered-spline model OR (95% CI)** | **Age and OR-duration centered-spline model OR (95% CI)** |
| --- | --- | --- | --- |
| Male vs Female | 1.136 (0.797–1.619) | 1.174 (0.823–1.675) | 1.167 (0.817–1.665) |
| Intestinal resection/stoma vs low complexity | 0.899 (0.493–1.639) | 0.896 (0.491–1.634) | 0.821 (0.449–1.503) |
| Laparotomy/adhesiolysis vs low complexity | 1.789 (1.023–3.128) | 1.795 (1.027–3.136) | 1.752 (1.004–3.057) |
| Upper abdominal major surgery vs low complexity | 1.389 (0.614–3.140) | 1.461 (0.645–3.307) | 1.650 (0.733–3.712) |
| ASA I–II vs ASA III | 0.550 (0.305–0.993) | 0.548 (0.304–0.986) | 0.555 (0.309–0.996) |
| ASA IV+ vs ASA III | 4.661 (2.916–7.449) | 4.794 (2.998–7.665) | 4.797 (3.006–7.655) |

**Note:** All models were fitted in the primary complete-case cohort (n = 5,349; broad composite events = 147) using conventional maximum-likelihood logistic regression. The primary model specified age and operating-room duration as linear continuous covariates. In the age centered-spline model, age was represented using a restricted cubic spline with three degrees of freedom and a centering constraint, while operating-room duration remained linear. In the joint centered-spline model, both age and operating-room duration were represented using centered restricted cubic spline terms with three degrees of freedom each. All models additionally included sex group, procedure group, and ASA physical status. Female sex, low-complexity common abdominal procedures, and ASA III were the reference categories. Individual spline-basis coefficients are not presented because they are not clinically interpretable in isolation. Likelihood-ratio tests comparing the continuous-covariate specifications are reported in Part D. The principal ASA and procedure-group estimates remained materially similar across specifications.

Table S 20 Procedure group definitions and representative procedures

| **Procedure group** | **Definition** | **Representative procedures** |
| --- | --- | --- |
| Low-complexity common abdominal procedures | Common lower complexity abdominal operations | Cholecystectomy, hernia repair, appendectomy |
| Intestinal resection/stoma | Bowel-related resection or reconstruction | Small bowel resection, colectomy, stoma creation |
| Laparotomy/adhesiolysis | Open exploration or adhesiolysis-intensive procedures | Exploratory laparotomy, adhesiolysis |
| Upper abdominal major surgery | Complex upper abdominal organ procedures | Gastrectomy, pancreatic resection/Whipple procedure, splenectomy |
