# Supplementary material for: Routinely available perioperative variables and EHR-captured postoperative adverse-event indicators after adult abdominal surgery: A retrospective cohort study using MOVER EPIC
Source: PLoS One. 2026 Oct 21;21(10):e0350704. doi: 10.1371/journal.pone.0350704 (PMC13686507; doi:10.1371/journal.pone.0350704)
Supplement: Supplementary file 2 [file pone.0350704.s003.docx]

**Response to the Academic Editor and Reviewers**

Manuscript ID: PONE-D-26-24069

Manuscript title: Routinely available perioperative variables and EHR-captured postoperative adverse-event indicators after adult abdominal surgery: a retrospective cohort study using MOVER EPIC

**Dear Dr. Subramanian and Reviewers,**

We sincerely thank the Academic Editor and the reviewers for their careful and constructive evaluation of our manuscript. We have revised the manuscript extensively in response to all comments. The revisions include expanded outcome definitions, more transparent documentation of the procedure-group classification, additional model-stability and diagnostic assessments, further sensitivity analyses addressing complete-case selection and repeated encounters, and an expanded discussion of the excluded encounters and the limitations of the EHR-derived outcomes.

All comments are reproduced below, followed by our point-by-point responses and a description of the corresponding changes in the revised manuscript.

Important correction identified during revision

During the revision, we conducted a systematic reproducibility audit of the cohort-construction and analysis code. This audit identified an error in the earlier text-based derivation of the ASA groups. Specifically, raw ASA_RATING_C code 4 (“Incapacitating Disease”) and code 6 (“Brain Dead”) had not been assigned to the ASA IV+ group. We corrected the derivation by reconstructing ASA groups directly from the numeric ASA_RATING_C field: codes 1–2 were classified as ASA I–II, code 3 as ASA III, and codes 4–6 as ASA IV+.

The source data, adult cohort definition, and outcome definitions were unchanged. The adult analytic cohort remained 5,679 encounters, with 166 broad-composite events and 95 narrow severe-composite events. However, because the earlier mapping error had incorrectly treated encounters with ASA codes 4 and 6 as analytically unavailable, correcting the mapping increased the primary complete-case cohort from 4,798 to 5,349 encounters. The number of broad-composite events in the primary cohort increased from 94 to 147, and the number of narrow severe-composite events increased from 49 to 84.

We reran all analyses involving ASA or the primary complete-case cohort, including the descriptive analyses, univariable and multivariable models, Firth penalized regression, patient-level cluster-robust inference, inverse-probability weighting, first-encounter sensitivity analysis, restricted cubic spline assessments, sparse-cell checks, and model diagnostics. We also performed an additional sensitivity analysis excluding the two encounters coded as ASA VI.

The corrected estimate for ASA IV+ versus ASA III in the primary broad-outcome model was an adjusted odds ratio of 4.66 (95% CI 2.92–7.45), compared with 6.86 (95% CI 3.50–13.42) in the earlier analysis. ASA IV+ therefore remained the strongest and most consistent correlate of the broad composite, although its estimated magnitude was attenuated. Some secondary estimates, particularly those involving procedure groups, also changed and have been revised and interpreted more cautiously throughout the manuscript.

We apologize for the earlier mapping error. The correction, its numerical consequences, and all updated analyses have been documented transparently in the revised manuscript, supplementary information, response letter, and updated reproducibility repository (<https://doi.org/10.5281/zenodo.21883349>).

**Academic Editor Comment 1**

“Outcome definitions clarifications.”

Response:

Thank you for highlighting the need for clearer outcome definitions. We have substantially expanded the “Outcome definitions” subsection of the Methods to distinguish the purpose, composition, and interpretation of the broad and narrow EHR-captured postoperative adverse-event indicator composites.

The primary broad composite was defined as a case-level indicator of whether at least one higher-level postoperative adverse-event category was recorded for the indexed surgical encounter. It included cardiovascular, respiratory, airway, neurological, metabolic, injury/infection, and death-related categories. We now clarify that this composite was designed to maximize the capture of postoperative morbidity signals available in MOVER EPIC and was not intended to represent a uniformly severe, clinically adjudicated, or standardized 30-day postoperative complication endpoint.

The secondary narrow composite was restricted to specific event-level indicators more directly reflecting major physiological deterioration or unplanned escalation of postoperative care. These included hemodynamic instability, hypotension, arrhythmia, cardiac arrest, hypoxemia, aspiration, hypercapnia, unplanned postoperative ventilation, reintubation, unplanned ICU admission recorded as a postoperative adverse-event item, and death. We clarify that the narrow composite was used as a supportive, sensitivity-oriented outcome and should not be interpreted as a formal Clavien–Dindo classification.

We also clarified that encounters with multiple component events contributed only once to each corresponding composite. Death was included in both composites when recorded and was additionally reported separately for transparency. Furthermore, unplanned ICU admission was included in the narrow composite only when explicitly recorded as a postoperative adverse-event item; administrative ICU indicators were not treated as outcomes because they could represent planned postoperative monitoring or resource use.

Detailed operational definitions and analytical roles are now provided in Supplementary Table S3. Component-specific inclusion rationales are provided in Supplementary Table S4a, and component-level frequencies in the final adult analytic cohort are reported in Supplementary Table S4b. The Abstract, Methods, Results, and Discussion have also been revised consistently to emphasize that these outcomes represent database-captured adverse-event indicators within the indexed perioperative encounter rather than standardized 30-day postoperative complication rates.

Changes made:

- Methods, Section 3.3, “Outcome definitions”
- Supplementary Table S3
- Supplementary Tables S4a and S4b
- Corresponding clarifications in the Abstract, Results, and Discussion

**Academic Editor Comment 2**

“Procedure group classifications - 4. Is this appropriate enough. Please expand on it. But I do understand that this helps in statistical analysis.”

Response:

Thank you for this important comment. We agree that the rationale and construction of the four procedure groups required greater transparency. We have expanded the Methods to explain that this classification was developed as an analytical grouping for the MOVER EPIC cohort rather than as a validated surgical taxonomy or an ordinal measure of operative risk.

The source procedure categories were consolidated into four groups to preserve clinically interpretable differences in operative anatomy and general procedural complexity while avoiding excessively sparse categories in the regression models. The four groups were defined as follows:

1. Low-complexity common abdominal procedures, including appendectomy, cholecystectomy, and hernia repair;
2. Intestinal resection/stoma procedures, including colorectal resection, small-bowel resection, and stoma surgery;
3. Laparotomy/adhesiolysis, including open abdominal exploration and adhesiolysis-intensive procedures; and
4. Upper abdominal major surgery, including gastrectomy, pancreatic surgery, and splenic procedures.

Laparotomy/adhesiolysis was retained as a separate group because these procedures may reflect open exploration, complex reoperative surgery, or substantial adhesiolysis. Gastrectomy, pancreatic surgery, and splenic procedures were grouped as upper abdominal major surgery because they involve complex upper abdominal organ procedures and would otherwise have remained within a heterogeneous residual category.

We now explicitly state that these groups were intended to support clinically interpretable regression analyses and were not intended to imply uniform operative severity within each group. We have therefore interpreted procedure-group estimates cautiously, particularly because several procedure-group associations were attenuated after ASA adjustment or were sensitive to alternative model specifications.

The revised main manuscript now provides a brief description and representative procedures for each group. Supplementary Table S6 presents the complete mapping from the original procedure categories to the four analytical groups, and Supplementary Table S20 provides the clinical definitions and representative procedures. The reproducibility repository also contains the procedure-group mapping file used in cohort construction.

Changes made:

- Methods, Section 3.4, “Candidate predictors and covariates”
- Supplementary Table S6
- Supplementary Table S20
- Corresponding cautionary interpretation in the Results and Discussion
- Updated procedure-group mapping file in the reproducibility repository

**Academic Editor Comment 3**

“With small number of outcome events, provide more info on model stability and diagnostic assessments.”

Response:

Thank you for this important comment. We have expanded the statistical methods, results, and supplementary information to provide detailed model-stability and diagnostic assessments for both the primary broad-composite model and the secondary narrow-outcome model.

Both multivariable models contained eight estimated parameters, excluding the intercept. In the corrected primary complete-case cohort, the broad-composite model included 147 events, corresponding to 18.4 events per parameter. The narrow severe-composite model included 84 events, corresponding to 10.5 events per parameter.

We assessed sparse categorical cells, model convergence, complete and quasi-complete separation, and multicollinearity. No predictor category had zero events or zero non-events. The minimum number of events in a categorical level was 12 for the broad model and 3 for the narrow model. Maximum-likelihood estimation converged successfully for both outcomes, and neither complete nor quasi-complete separation was detected. The maximum variance inflation factor was 1.84, indicating no evidence of problematic multicollinearity.

Because the lower number of events, particularly in the narrow model, could increase small-sample bias in conventional maximum-likelihood estimates, we performed Firth penalized logistic regression for both outcomes. Both Firth models converged successfully, and the direction and magnitude of the principal associations remained broadly similar to the maximum-likelihood estimates. We also assessed the robustness of the primary model using patient-level cluster-robust standard errors, restriction to the first eligible encounter per patient, and inverse-probability weighting for complete-case selection.

We have added these diagnostic results to Supplementary Table S19. We also explicitly state that, although the narrow model met an events-per-parameter ratio of 10.5 and showed no evidence of separation or convergence failure, it contained fewer events and a minimum of only three events in one categorical level. Accordingly, the narrow-outcome analysis continues to be interpreted as supportive and exploratory rather than definitive.

Changes made:

- Methods, Section 3.7, “Statistical analysis”
- Results, primary model-stability assessment
- Results, secondary narrow-outcome analysis
- Supplementary Table S19
- Corresponding cautionary interpretation in the Discussion

**Academic Editor Comment 4**

“Provide more detail on excluded patients and its impact.”

Response:

Thank you for this important comment. We have expanded the description of the encounters excluded from the primary complete-case analysis and evaluated the potential impact of complete-case selection. Of the 5,679 encounters in the deduplicated adult analytic cohort, 330 (5.8%) were excluded from the primary regression cohort because of missing ASA physical status or operating-room duration. Among these encounters, 275 were missing both variables, 16 were missing ASA only, and 39 were missing operating-room duration only.

Compared with included encounters, excluded encounters had a higher frequency of the broad composite (5.76% versus 2.75%; p = 0.003), a longer median hospital length of stay (5 versus 3 days; p < 0.001), and a greater representation of laparotomy/adhesiolysis procedures (39.39% versus 21.95%; overall procedure-group p < 0.001). These differences suggest that complete-case selection may have under-represented encounters with greater clinical complexity or postoperative morbidity.

We therefore performed an inverse-probability-weighted sensitivity analysis. Among the 5,678 encounters with Female or Male sex coding, 5,349 were complete cases and 329 were non-complete cases. Stabilized weights showed good overlap, preserved nearly the full effective sample size, and improved measured covariate balance. The weighted estimates were materially similar to the primary complete-case estimates, although the confidence interval for ASA I–II included 1. We have therefore retained the primary complete-case analysis while adding a more cautious discussion of selection bias. We also acknowledge that IPW addresses selection conditional on measured variables and cannot eliminate bias related to unmeasured determinants of missingness.

Changes made:

- Methods, Section 3.7
- Results, comparison of included and excluded encounters
- Results, inverse-probability-weighted sensitivity analysis
- Discussion, limitations of complete-case selection
- Supplementary Tables S15 and S19

**Reviewer #1**

Comment:

“The study is methodologically sound, with the appropriate statistical analyses applied to both continuous and categorical variables. The use of descriptive statistics, regression analyses, and odds ratio is appropriate for the study objectives, and the conclusions are well supported by the presented results.”

Response:

We sincerely thank Reviewer #1 for the positive assessment of the methodological approach, statistical analyses, and interpretation of the findings. Although no specific revisions were requested by Reviewer #1, we have extensively revised the manuscript in response to the Academic Editor and Reviewer #2. These revisions include clearer outcome definitions, expanded documentation of the procedure-group classification, additional model diagnostics and sensitivity analyses, a detailed evaluation of complete-case selection, and more cautious interpretation of the EHR-derived outcomes and clinician-assigned ASA physical status.

**Reviewer #2, Comment 1**

“First, the outcome definitions require additional clarification. The primary and secondary outcomes are composites of EHR-captured adverse-event indicators, but these are not externally adjudicated complications and do not have a standardized 30-day follow-up window. The authors acknowledge this limitation, which is important. However, the manuscript would be stronger if the authors provided more explicit justification for each component included in the broad and narrow composites. For example, some broad categories may represent heterogeneous clinical events with very different severities.”

Response:

Thank you for this important observation. We agree that the components of the broad composite are clinically heterogeneous and should not be interpreted as events of uniform severity. We have therefore expanded the outcome definitions and added component-specific inclusion rationales.

The broad composite was constructed from the higher-level postoperative adverse-event categories available in MOVER EPIC, including cardiovascular, respiratory, airway, neurological, metabolic, injury/infection, and death-related events. These categories were retained to maximize the capture of postoperative morbidity signals recorded in the database. We now state explicitly that individual events within these categories may differ substantially in clinical severity and that the broad composite is neither a severity-graded endpoint nor an externally adjudicated complication measure.

The narrow composite was restricted to specific event-level indicators more directly representing major physiological deterioration or unplanned escalation of care. These included hemodynamic instability, hypotension, arrhythmia, cardiac arrest, hypoxemia, aspiration, hypercapnia, unplanned postoperative ventilation, reintubation, unplanned ICU admission recorded as a postoperative adverse-event item, and death. Administrative ICU indicators were excluded because they may represent planned postoperative monitoring or resource utilization rather than an adverse event.

Supplementary Table S4a now provides the clinical interpretation and explicit inclusion rationale for every component of both composites. Supplementary Table S4b reports the frequency of each component in the final adult analytic cohort. We also clarify that component counts are not mutually exclusive because one encounter could contain more than one recorded event, although each encounter contributed only once to the corresponding case-level composite.

Throughout the revised manuscript, we consistently describe these outcomes as “EHR-captured postoperative adverse-event indicator composites.” We emphasize that they reflect events captured within the indexed perioperative encounter and should not be interpreted as standardized 30-day postoperative complication rates or directly compared with registries using adjudicated definitions and standardized follow-up.

Changes made:

- Methods, Section 3.3, “Outcome definitions”
- Supplementary Table S3
- Supplementary Tables S4a and S4b
- Clarifying language in the Abstract, Results, and Discussion
- Expanded limitations concerning outcome heterogeneity, ascertainment, and the absence of standardized 30-day follow-up

**Reviewer #2, Comment 2**

“Second, the event rate is low. The broad composite occurred in 166 of 5,679 encounters, and the primary complete-case model included only 94 broad events. The narrow severe outcome included only 49 events in the complete-case cohort. The authors appropriately describe the narrow analysis as exploratory, but the low number of events limits the stability of the estimates, especially for categorical predictors with multiple levels. Please provide additional model diagnostics, such as the number of events per modeled parameter, assessment of sparse cells, convergence diagnostics, and whether penalized logistic regression was considered as a sensitivity analysis.”

Response:

Thank you for raising this important concern. As described in the “Important correction identified during revision” section of this response letter, the reproducibility audit identified that raw ASA codes 4 and 6 had been omitted from the earlier text-based ASA IV+ derivation. The overall adult cohort and outcome counts were unchanged, but correction of the ASA mapping increased the primary complete-case cohort from 4,798 to 5,349 encounters. Consequently, the number of broad-composite events in the primary model increased from 94 to 147, and the number of narrow severe-composite events increased from 49 to 84.

We nevertheless agree that the event counts, particularly for the narrow outcome, require explicit assessment of model stability. Both multivariable models contained eight estimated parameters, excluding the intercept. The corrected broad model therefore had 18.4 events per parameter, while the narrow model had 10.5 events per parameter.

We examined the categorical predictor levels for sparse event and non-event cells. No category had zero events or zero non-events. The minimum number of events in a categorical level was 12 for the broad outcome and 3 for the narrow outcome. Both maximum-likelihood models converged successfully, and neither complete nor quasi-complete separation was detected. The maximum variance inflation factor was 1.84, indicating no evidence of problematic multicollinearity.

In direct response to the reviewer’s suggestion, we performed Firth penalized logistic regression as a sensitivity analysis for both the broad and narrow outcomes. Both Firth models converged successfully. The direction and magnitude of the principal associations, particularly those involving clinician-assigned ASA physical status, remained broadly similar to the conventional maximum-likelihood estimates. We also performed patient-level cluster-robust inference and a first-encounter-per-patient sensitivity analysis to address repeated encounters.

These assessments are now reported in the Statistical Analysis and Results sections and summarized in Supplementary Table S19. Despite the successful convergence, absence of separation, and broadly consistent Firth estimates, we continue to describe the narrow-outcome model as supportive and exploratory because of its lower event count and the presence of a categorical level containing only three events.

Changes made:

- Updated all model-specific cohort and event counts
- Methods, Section 3.7, “Statistical analysis”
- Results, primary model-stability assessment
- Results, secondary narrow-outcome analysis
- Supplementary Tables S8b and S19
- Expanded cautionary interpretation of the narrow model

**Reviewer #2, Comment 3**

“Third, the complete-case approach may introduce selection bias. The manuscript states that excluded encounters had a higher frequency of captured broad adverse-event indicators, longer hospital stays, and a greater proportion of laparotomy/adhesiolysis procedures. This is important because it suggests that the primary complete-case cohort may under-represent higher-risk cases. The ASA-excluded sensitivity analysis is helpful, but the authors should expand the discussion of how this missingness could affect the direction and magnitude of the results. If feasible, multiple imputation or inverse-probability weighting could be considered as an additional sensitivity analysis. If not feasible, the rationale should be explained.”

Response:

Thank you. We agree that the higher broad-composite frequency, longer length of stay, and greater representation of laparotomy/adhesiolysis among excluded encounters required a more direct assessment of potential complete-case selection bias. We selected inverse probability weighting, one of the approaches suggested by the reviewer, because it allowed us to assess the sensitivity of the primary complete-case estimates to selection conditional on measured variables without imputing the missing ASA or operating-room-duration values. Among the 5,678 encounters with Female or Male sex coding, we modeled the probability of inclusion in the primary complete-case cohort using age, sex group, procedure group, the broad composite outcome, log-transformed hospital length of stay and log-transformed anesthesia duration, each with a missingness indicator, patient class, and ICU admission. We calculated stabilized weights and refitted the primary outcome model using sandwich-robust standard errors. The weights showed good overlap (mean 1.000, SD 0.035; range 0.949–1.178), no weight exceeded 1.25, and the effective sample size was 5,342.6. Measured covariate balance improved from a maximum absolute standardized mean difference of 0.024 before weighting to 0.001 after weighting. The IPW estimates were materially similar to the primary estimates for ASA IV+ (OR 4.78, 95% CI 3.06–7.48) and laparotomy/adhesiolysis (OR 1.81, 95% CI 1.02–3.19). The ASA I–II point estimate was essentially unchanged, but its robust confidence interval included 1 (OR 0.55, 95% CI 0.29–1.03), and we have softened the interpretation of this association accordingly. Results were also materially unchanged after truncation at the 1st and 99th percentiles. These additions are now reported in Methods Section 3.7, the Results model-stability paragraph, the Discussion limitations section, and Supplementary Table S19. We also note that IPW addresses selection conditional on measured variables and cannot exclude bias related to unmeasured determinants of missingness.

Changes made:

- Methods, Sections 3.6 and 3.7
- Results, comparison of included and excluded encounters
- Inverse-probability-weighted sensitivity analyses
- Supplementary Tables S15 and S19
- Expanded discussion of the possible direction and magnitude of complete-case selection bias
- Aggregate IPW estimates and weight diagnostics added to the reproducibility repository

**Reviewer #2, Comment 4**

“Fourth, the modeling strategy would benefit from more assessment of assumptions. Age and operating-room duration were modeled as continuous variables, but only operating-room duration appears to have been examined categorically in sensitivity analysis. Please consider assessing nonlinearity for both age and operating-room duration, for example using restricted cubic splines or clinically meaningful categories. In addition, because repeated encounters from the same patient were possible, the first-encounter sensitivity analysis is useful, but the authors should also consider clustered standard errors or a mixed-effects model if patient identifiers allow this.”

Response:

Thank you for these helpful suggestions. We have added analyses addressing both the functional-form assumptions for the continuous covariates and the potential within-patient correlation arising from repeated surgical encounters.

First, we assessed potential nonlinearity in age and operating-room duration using centered restricted cubic spline terms with three degrees of freedom. Each spline model was compared with the corresponding linear specification using a likelihood-ratio test. The age spline model showed modestly improved fit compared with the linear age specification (likelihood-ratio χ² = 6.39, df = 2; p = 0.041). The corresponding evidence for operating-room duration was weaker and did not reach statistical significance (χ² = 4.91, df = 2; p = 0.086). A model in which both age and operating-room duration were represented using centered spline terms also improved fit compared with the model containing both linear terms (χ² = 11.11, df = 4; p = 0.025).

We therefore revised the manuscript to acknowledge modest evidence of nonlinearity in the association between age and the broad composite. Importantly, the principal adjusted estimates remained materially unchanged under the alternative spline specifications. In the age-spline model, the adjusted OR was 4.79 (95% CI 3.00–7.67) for ASA IV+ versus ASA III, 0.55 (95% CI 0.30–0.99) for ASA I–II versus ASA III, and 1.79 (95% CI 1.03–3.14) for laparotomy/adhesiolysis versus low-complexity procedures. The corresponding estimates also remained similar when both continuous covariates were modeled using centered spline terms, as shown in Supplementary Table S19, Part E.

Because the purpose of these analyses was to assess sensitivity to the continuous-covariate functional form, and because the principal ASA and procedure-group estimates were materially unchanged, we retained the originally specified linear formulation as the primary parsimonious model and present the centered spline models as sensitivity analyses. However, we no longer state that the relationship with age was adequately characterized by a single linear term, and we caution against interpreting the per-year age odds ratio as a complete summary of the age–outcome relationship.

Second, because patient identifiers were available and some patients contributed more than one eligible encounter, we refitted the primary model using patient-level cluster-robust standard errors. This approach retained the surgical encounter as the analytical unit while accounting for within-patient correlation. The point estimates were unchanged, and the overall inference remained similar. ASA IV+ remained strongly associated with the broad composite, whereas the weaker laparotomy/adhesiolysis association became borderline under cluster-robust inference (OR 1.79, 95% CI 0.99–3.23; p = 0.053).

We also retained only the earliest eligible encounter for each patient and refitted the primary model. This first-encounter analysis included 4,704 encounters and 108 broad-composite events. ASA IV+ remained strongly associated with the outcome (OR 4.67, 95% CI 2.72–8.01), and the overall pattern of estimates remained consistent with the primary analysis.

We selected cluster-robust inference and restriction to the first eligible encounter as complementary approaches because the study objective concerned marginal encounter-level associations rather than estimation of patient-specific random effects. Together, these analyses addressed within-patient correlation without requiring assumptions concerning the distribution of a patient-level random intercept.

Changes made:

- Methods, Section 3.7, “Statistical analysis”
- Abstract and Results, interpretation of the age and operating-room-duration functional forms
- Supplementary Table S19, Parts D and E
- Results, patient-level cluster-robust and first-encounter analyses
- Supplementary Tables S16 and S17

**Reviewer #2, Comment 5**

“Fifth, the procedure-group classification should be described more transparently in the main manuscript. The grouping into low-complexity common abdominal procedures, intestinal resection/stoma, upper abdominal major surgery, and laparotomy/adhesiolysis is clinically reasonable, but the reader needs to understand what specific procedures were included in each group. The authors refer to supplementary tables, but a brief summary in the main text would improve interpretability.”

Response:

Thank you for this helpful suggestion. We agree that readers should be able to understand the four procedure groups from the main manuscript without relying exclusively on the supplementary information.

We have expanded the Methods to provide a brief definition and representative procedures for each group. Specifically:

1. Low-complexity common abdominal procedures included appendectomy, cholecystectomy, and hernia repair;
2. Intestinal resection/stoma procedures included colorectal resection, small-bowel resection, and stoma surgery;
3. Laparotomy/adhesiolysis included open abdominal exploration and adhesiolysis-intensive procedures; and
4. Upper abdominal major surgery included gastrectomy, pancreatic surgery, and splenic procedures.

We also clarify in the main manuscript that these groups were created to preserve clinically interpretable differences in operative anatomy and general complexity while avoiding sparse procedure categories in the regression models. The classification was intended as a study-specific analytical grouping rather than a validated surgical taxonomy or an ordinal severity scale.

Supplementary Table S6 provides the complete mapping from the original procedure categories to the four analytical groups. Supplementary Table S20 provides concise definitions and representative procedures, and the reproducibility repository includes the procedure-group mapping file applied during cohort construction.

Changes made:

- Expanded procedure-group description in Methods, Section 3.4
- Supplementary Table S6
- Supplementary Table S20
- Procedure-group mapping file in the reproducibility repository

**Reviewer #2, Comment 6**

“Sixth, the interpretation of ASA physical status should be cautious. ASA is a pragmatic clinical summary and is strongly associated with postoperative outcomes, but it is subjective and may incorporate clinician judgment, comorbidity burden, urgency, and other unmeasured risk factors. The manuscript appropriately notes this, but some parts of the discussion could overstate ASA as a direct marker of ‘baseline physiological vulnerability.’ I suggest consistently using language such as ‘clinician-assigned ASA physical status, which may reflect comorbidity burden and perceived perioperative vulnerability.’”

Response:

Thank you for this important clarification. We agree that clinician-assigned ASA physical status should not be interpreted as a direct or objective measurement of baseline physiological vulnerability, physiological reserve, or frailty.

We have revised the Abstract, Results, Discussion, and Conclusion to describe ASA consistently as a pragmatic, clinician-assigned clinical classification. We now state that it may reflect a combination of documented comorbidity burden, functional status, clinician judgment, perceived perioperative vulnerability, and other measured or unmeasured clinical information.

We have removed or revised wording that could imply that ASA directly measures physiological reserve or that the observed associations represent a causal effect of ASA classification. The revised Discussion emphasizes that ASA is partly subjective and may summarize clinical information that was not otherwise available as separate covariates in the MOVER EPIC dataset. Consequently, the ASA estimates may reflect residual confounding by underlying health status, comorbidity burden, functional limitation, urgency, or other unmeasured perioperative risk factors.

We have also revised our interpretation of the attenuation of procedure-group estimates after ASA adjustment. Rather than suggesting that ASA independently represents biological vulnerability, we now state that some operative-context information may overlap with the broader clinical risk context reflected in clinician-assigned ASA physical status.

The revised Conclusion therefore describes ASA as the most consistent correlate of the database-captured adverse-event indicators while explicitly stating that it should not be interpreted as a direct measure of physiological reserve or as an independently causal determinant of postoperative morbidity.

Changes made:

- Revised ASA terminology in the Abstract
- Revised interpretation of ASA in the Results and Discussion
- Removed wording implying direct measurement of physiological vulnerability or reserve
- Expanded discussion of subjectivity, clinician judgment, residual confounding, and unmeasured clinical information
- Revised Conclusion
