# Supplementary material for: Routinely available perioperative variables and EHR-captured postoperative adverse-event indicators after adult abdominal surgery: A retrospective cohort study using MOVER EPIC
Source: PLoS One. 2026 Oct 21;21(10):e0350704. doi: 10.1371/journal.pone.0350704 (PMC13686507; doi:10.1371/journal.pone.0350704)
Supplement: Peer review history

# Peer review: Decision Letter 0

**DOI:** 10.1371/journal.pone.0350704.r001

**Authors:** Jayaram Subramanian

6 Aug 2026

Routinely available perioperative variables and EHR-captured postoperative adverse-event indicators after adult abdominal surgery: a retrospective cohort study using MOVER EPIC

PLOS One

Dear Dr. Cui,

Thank you for submitting your manuscript to PLOS ONE. After careful consideration, we feel that it has merit but does not fully meet PLOS ONE’s publication criteria as it currently stands. Therefore, we invite you to submit a revised version of the manuscript that addresses the points raised during the review process.

==============================

**ACADEMIC EDITOR:**

Please clarify these points in your paper.

1. Outcome definitions clarifications.

2. Procedure group classifications - 4. Is this appropriate enough. Please expand on it. But i do understand that this helps in statistical analysis.

3. With small number of outcome events, provide more info on model stability and diagnostic assessments.

4. Provide more detail on excluded patients and its impact.

==============================

Please submit your revised manuscript by Sep 20 2026 11:59PM. If you will need more time than this to complete your revisions, please reply to this message or contact the journal office at plosone@plos.org. When you're ready to submit your revision, log on to https://www.editorialmanager.com/pone/ and select the 'Submissions Needing Revision' folder to locate your manuscript file.

- A letter that responds to each point raised by the academic editor and reviewer(s). You should upload this letter as a separate file labeled 'Response to Reviewers'.
- A marked-up copy of your manuscript that highlights changes made to the original version. You should upload this as a separate file labeled 'Revised Manuscript with Track Changes'.
- An unmarked version of your revised paper without tracked changes. You should upload this as a separate file labeled 'Manuscript'.

If you would like to make changes to your financial disclosure, please include your updated statement in your cover letter. Guidelines for resubmitting your figure files are available below the reviewer comments at the end of this letter.

If applicable, we recommend that you deposit your laboratory protocols in protocols.io to enhance the reproducibility of your results. Protocols.io assigns your protocol its own identifier (DOI) so that it can be cited independently in the future. For instructions see: https://journals.plos.org/plosone/s/submission-guidelines#loc-laboratory-protocols. Additionally, PLOS ONE offers an option for publishing peer-reviewed Lab Protocol articles, which describe protocols hosted on protocols.io. Read more information on sharing protocols at https://plos.org/protocols?utm_medium=editorial-email&utm_source=authorletters&utm_campaign=protocols

As the corresponding author, your ORCID iD is verified in the submission system and will appear in the published article. PLOS supports the use of ORCID, and we encourage all coauthors to register for an ORCID iD and use it as well. Please encourage your coauthors to verify their ORCID iD within the submission system before final acceptance, as unverified ORCID iDs will not appear in the published article. *Only*  the individual author can complete the verification step; PLOS staff *cannot*  verify ORCID iDs on behalf of authors.

We look forward to receiving your revised manuscript.

Kind regards,

Jayaram Subramanian, Ph.D.

Academic Editor

PLOS One

Journal Requirements:

When submitting your revision, we need you to address these additional requirements.

1. Please ensure that your manuscript meets PLOS ONE's style requirements, including those for file naming. The PLOS ONE style templates can be found at https://journals.plos.org/plosone/s/file?id=wjVg/PLOSOne_formatting_sample_main_body.pdf and https://journals.plos.org/plosone/s/file?id=ba62/PLOSOne_formatting_sample_title_authors_affiliations.pdf

2. Thank you for your submission to PLOS ONE. We note that you have declared the use of generative AI in the preparation of your manuscript in the cover letter. Before we can proceed, please report the version of ChatGPT used in preparing this manuscript. More information about our AI policy can be found here: (https://journals.plos.org/plosone/s/ethical-publishing-practice#loc-artificial-intelligence-tools-and-technologies).

3. Thank you for stating the following in the Competing Interests section:

[I have read the journal's policy and the authors of this manuscript have the following potential competing interests: Xinyue Cui is affiliated with Sichuan Global Zongsen Tanke Technology Co., Ltd. This affiliation did not provide external funding for the study and does not represent a commercial interest in the MOVER database, the study topic, the analysis, or the publication of the findings. The authors declare no other competing interests.].

We note that one or more of the authors are employed by a commercial company Sichuan Global Zongsen Tanke Technology Co., Ltd.

1. Please provide an amended Funding Statement declaring this commercial affiliation, as well as a statement regarding the Role of Funders in your study. If the funding organization did not play a role in the study design, data collection and analysis, decision to publish, or preparation of the manuscript and only provided financial support in the form of authors' salaries and/or research materials, please review your statements relating to the author contributions, and ensure you have specifically and accurately indicated the role(s) that these authors had in your study. You can update author roles in the Author Contributions section of the online submission form.

Please also include the following statement within your amended Funding Statement.

“The funder provided support in the form of salaries for authors [insert relevant initials], but did not have any additional role in the study design, data collection and analysis, decision to publish, or preparation of the manuscript. The specific roles of these authors are articulated in the ‘author contributions’ section.”

If your commercial affiliation did play a role in your study, please state and explain this role within your updated Funding Statement.

2. Please also provide an updated Competing Interests Statement declaring this commercial affiliation along with any other relevant declarations relating to employment, consultancy, patents, products in development, or marketed products, etc.

Within your Competing Interests Statement, please confirm that this commercial affiliation does not alter your adherence to all PLOS ONE policies on sharing data and materials by including the following statement: "This does not alter our adherence to PLOS ONE policies on sharing data and materials.” (as detailed online in our guide for authors http://journals.plos.org/plosone/s/competing-interests). If this adherence statement is not accurate and there are restrictions on sharing of data and/or materials, please state these. Please note that we cannot proceed with consideration of your article until this information has been declared.

Please include both an updated Funding Statement and Competing Interests Statement in your cover letter. We will change the online submission form on your behalf.

4. We note you have included a table to which you do not refer in the text of your manuscript. Please ensure that you refer to Table 1 in your text; if accepted, production will need this reference to link the reader to the Table.

5. Please include captions for your Supporting Information files at the end of your manuscript, and update any in-text citations to match accordingly. Please see our Supporting Information guidelines for more information: http://journals.plos.org/plosone/s/supporting-information.

If the reviewer comments include a recommendation to cite specific previously published works, please review and evaluate these publications to determine whether they are relevant and should be cited. There is no requirement to cite these works unless the editor has indicated otherwise.

Please review your reference list to ensure that it is complete and correct. If you have cited papers that have been retracted, please include the rationale for doing so in the manuscript text, or remove these references and replace them with relevant current references. Any changes to the reference list should be mentioned in the rebuttal letter that accompanies your revised manuscript. If you need to cite a retracted article, indicate the article’s retracted status in the References list and also include a citation and full reference for the retraction notice.

Additional Editor Comments:

The topic addressed in the paper is important. Study in general is conducted well and the researchers have shown the limitation of using EHR derived indicators. Findings are relevant and reasonable. Limitations are mentioned in the paper for the readers to understand clearly.

Some clarifications are needed

1. Outcome definitions clarifications.

2. Procedure group classifications - 4. Is this appropriate enough. Please expand on it. But i do understand that this helps in statistical analysis.

3. With small number of outcome events, provide more info on model stability and diagnostic assessments.

4. Provide more detail on excluded patients and its impact.

Overall it is a good paper but needs to address the clarifications on some of the limitations and statistical decisions taken.

[Note: HTML markup is below. Please do not edit.]

Reviewers' comments:

Reviewer's Responses to Questions

**Comments to the Author**

1. Is the manuscript technically sound, and do the data support the conclusions?

Reviewer #1: Yes

Reviewer #2: Partly

**********

2. Has the statistical analysis been performed appropriately and rigorously?

Reviewer #1: Yes

Reviewer #2: Yes

**********

3. Have the authors made all data underlying the findings in their manuscript fully available?

The PLOS Data policy

Reviewer #1: Yes

Reviewer #2: Yes

**********

4. Is the manuscript presented in an intelligible fashion and written in standard English?

Reviewer #1: Yes

Reviewer #2: Yes

**********

Reviewer #1: yes, the study is methodologically sound, with the appropriate statistical analyses applied to both continuous and categorical variables. The use of descriptive statistics, regression analyses, and odds ratio is appropriate for the study objectives, and the conclusions are well supported by the presented results.

Reviewer #2: This retrospective cohort study examines routinely available perioperative variables associated with EHR-captured postoperative adverse-event indicators after adult abdominal surgery in the MOVER EPIC database. The study addresses an important methodological issue: adverse-event indicators available in perioperative EHR datasets may not correspond to standardized, adjudicated postoperative complication endpoints. The authors are appropriately cautious in stating that their results should not be interpreted as standardized 30-day postoperative complication rates.

Overall, the manuscript is potentially suitable for publication after revision. The study is clearly framed as exploratory, uses a public perioperative data source, and focuses on clinically interpretable variables rather than overfitting a prediction model. The main finding—that ASA physical status is the most consistent correlate of database-captured postoperative adverse-event indicators—is plausible and supported by the analyses presented. The sensitivity analyses excluding ASA, categorizing operating-room duration, and restricting to the first eligible encounter per patient are also useful.

However, several issues should be addressed before publication.

First, the outcome definitions require additional clarification. The primary and secondary outcomes are composites of EHR-captured adverse-event indicators, but these are not externally adjudicated complications and do not have a standardized 30-day follow-up window. The authors acknowledge this limitation, which is important. However, the manuscript would be stronger if the authors provided more explicit justification for each component included in the broad and narrow composites. For example, some broad categories may represent heterogeneous clinical events with very different severities.

Second, the event rate is low. The broad composite occurred in 166 of 5,679 encounters, and the primary complete-case model included only 94 broad events. The narrow severe outcome included only 49 events in the complete-case cohort. The authors appropriately describe the narrow analysis as exploratory, but the low number of events limits the stability of the estimates, especially for categorical predictors with multiple levels. Please provide additional model diagnostics, such as the number of events per modeled parameter, assessment of sparse cells, convergence diagnostics, and whether penalized logistic regression was considered as a sensitivity analysis.

Third, the complete-case approach may introduce selection bias. The manuscript states that excluded encounters had a higher frequency of captured broad adverse-event indicators, longer hospital stays, and a greater proportion of laparotomy/adhesiolysis procedures. This is important because it suggests that the primary complete-case cohort may under-represent higher-risk cases. The ASA-excluded sensitivity analysis is helpful, but the authors should expand the discussion of how this missingness could affect the direction and magnitude of the results. If feasible, multiple imputation or inverse-probability weighting could be considered as an additional sensitivity analysis. If not feasible, the rationale should be explained.

Fourth, the modeling strategy would benefit from more assessment of assumptions. Age and operating-room duration were modeled as continuous variables, but only operating-room duration appears to have been examined categorically in sensitivity analysis. Please consider assessing nonlinearity for both age and operating-room duration, for example using restricted cubic splines or clinically meaningful categories. In addition, because repeated encounters from the same patient were possible, the first-encounter sensitivity analysis is useful, but the authors should also consider clustered standard errors or a mixed-effects model if patient identifiers allow this.

Fifth, the procedure-group classification should be described more transparently in the main manuscript. The grouping into low-complexity common abdominal procedures, intestinal resection/stoma, upper abdominal major surgery, and laparotomy/adhesiolysis is clinically reasonable, but the reader needs to understand what specific procedures were included in each group. The authors refer to supplementary tables, but a brief summary in the main text would improve interpretability.

Sixth, the interpretation of ASA physical status should be cautious. ASA is a pragmatic clinical summary and is strongly associated with postoperative outcomes, but it is subjective and may incorporate clinician judgment, comorbidity burden, urgency, and other unmeasured risk factors. The manuscript appropriately notes this, but some parts of the discussion could overstate ASA as a direct marker of “baseline physiological vulnerability.” I suggest consistently using language such as “clinician-assigned ASA physical status, which may reflect comorbidity burden and perceived perioperative vulnerability.”

Finally, the manuscript is understandable and generally well written, but it is repetitive. The phrase “EHR-captured postoperative adverse-event indicator composite” appears very frequently and makes the text harder to read. The authors could define this term once and then use a shorter expression such as “broad EHR adverse-event composite” or “broad composite.” There are also minor grammar issues. For example, “frailty-related measure” should likely be “frailty-related measures” or “a frailty-related measure.” The sentence beginning “In the present study, the association between ASA IV+ and odds of EHR-captured adverse-event indicators after adjustment for chronological age...” is incomplete or awkward and should be revised.

**********

what does this mean?). If published, this will include your full peer review and any attached files.

If you choose “no”, your identity will remain anonymous but your review may still be made public.

**Do you want your identity to be public for this peer review?**  For information about this choice, including consent withdrawal, please see our Privacy Policy

Reviewer #1: No

Reviewer #2: **Yes:**  Yuri Chaves Martins

**********

[NOTE: If reviewer comments were submitted as an attachment file, they will be attached to this email and accessible via the submission site. Please log into your account, locate the manuscript record, and check for the action link "View Attachments". If this link does not appear, there are no attachment files.]

To ensure your figures meet our technical requirements, please review our figure guidelines: https://journals.plos.org/plosone/s/figures

You may also use PLOS’s free figure tool, NAAS, to help you prepare publication quality figures: https://journals.plos.org/plosone/s/figures#loc-tools-for-figure-preparation.

NAAS will assess whether your figures meet our technical requirements by comparing each figure against our figure specifications.

# Author comment: Author response to Decision Letter 1

**DOI:** 10.1371/journal.pone.0350704.r002

19 Aug 2026

Response to the Academic Editor and Reviewers

Manuscript ID: PONE-D-26-24069

Manuscript title: Routinely available perioperative variables and EHR-captured postoperative adverse-event indicators after adult abdominal surgery: a retrospective cohort study using MOVER EPIC

Dear Dr. Subramanian and Reviewers,

We sincerely thank the Academic Editor and the reviewers for their careful and constructive evaluation of our manuscript. We have revised the manuscript extensively in response to all comments. The revisions include expanded outcome definitions, more transparent documentation of the procedure-group classification, additional model-stability and diagnostic assessments, further sensitivity analyses addressing complete-case selection and repeated encounters, and an expanded discussion of the excluded encounters and the limitations of the EHR-derived outcomes.

All comments are reproduced below, followed by our point-by-point responses and a description of the corresponding changes in the revised manuscript.

Important correction identified during revision

During the revision, we conducted a systematic reproducibility audit of the cohort-construction and analysis code. This audit identified an error in the earlier text-based derivation of the ASA groups. Specifically, raw ASA_RATING_C code 4 (“Incapacitating Disease”) and code 6 (“Brain Dead”) had not been assigned to the ASA IV+ group. We corrected the derivation by reconstructing ASA groups directly from the numeric ASA_RATING_C field: codes 1–2 were classified as ASA I–II, code 3 as ASA III, and codes 4–6 as ASA IV+.

The source data, adult cohort definition, and outcome definitions were unchanged. The adult analytic cohort remained 5,679 encounters, with 166 broad-composite events and 95 narrow severe-composite events. However, because the earlier mapping error had incorrectly treated encounters with ASA codes 4 and 6 as analytically unavailable, correcting the mapping increased the primary complete-case cohort from 4,798 to 5,349 encounters. The number of broad-composite events in the primary cohort increased from 94 to 147, and the number of narrow severe-composite events increased from 49 to 84.

We reran all analyses involving ASA or the primary complete-case cohort, including the descriptive analyses, univariable and multivariable models, Firth penalized regression, patient-level cluster-robust inference, inverse-probability weighting, first-encounter sensitivity analysis, restricted cubic spline assessments, sparse-cell checks, and model diagnostics. We also performed an additional sensitivity analysis excluding the two encounters coded as ASA VI.

The corrected estimate for ASA IV+ versus ASA III in the primary broad-outcome model was an adjusted odds ratio of 4.66 (95% CI 2.92–7.45), compared with 6.86 (95% CI 3.50–13.42) in the earlier analysis. ASA IV+ therefore remained the strongest and most consistent correlate of the broad composite, although its estimated magnitude was attenuated. Some secondary estimates, particularly those involving procedure groups, also changed and have been revised and interpreted more cautiously throughout the manuscript.

We apologize for the earlier mapping error. The correction, its numerical consequences, and all updated analyses have been documented transparently in the revised manuscript, supplementary information, response letter, and updated reproducibility repository (https://doi.org/10.5281/zenodo.21883349).

Academic Editor Comment 1

“Outcome definitions clarifications.”

Response:

Thank you for highlighting the need for clearer outcome definitions. We have substantially expanded the “Outcome definitions” subsection of the Methods to distinguish the purpose, composition, and interpretation of the broad and narrow EHR-captured postoperative adverse-event indicator composites.

The primary broad composite was defined as a case-level indicator of whether at least one higher-level postoperative adverse-event category was recorded for the indexed surgical encounter. It included cardiovascular, respiratory, airway, neurological, metabolic, injury/infection, and death-related categories. We now clarify that this composite was designed to maximize the capture of postoperative morbidity signals available in MOVER EPIC and was not intended to represent a uniformly severe, clinically adjudicated, or standardized 30-day postoperative complication endpoint.

The secondary narrow composite was restricted to specific event-level indicators more directly reflecting major physiological deterioration or unplanned escalation of postoperative care. These included hemodynamic instability, hypotension, arrhythmia, cardiac arrest, hypoxemia, aspiration, hypercapnia, unplanned postoperative ventilation, reintubation, unplanned ICU admission recorded as a postoperative adverse-event item, and death. We clarify that the narrow composite was used as a supportive, sensitivity-oriented outcome and should not be interpreted as a formal Clavien–Dindo classification.

We also clarified that encounters with multiple component events contributed only once to each corresponding composite. Death was included in both composites when recorded and was additionally reported separately for transparency. Furthermore, unplanned ICU admission was included in the narrow composite only when explicitly recorded as a postoperative adverse-event item; administrative ICU indicators were not treated as outcomes because they could represent planned postoperative monitoring or resource use.

Detailed operational definitions and analytical roles are now provided in Supplementary Table S3. Component-specific inclusion rationales are provided in Supplementary Table S4a, and component-level frequencies in the final adult analytic cohort are reported in Supplementary Table S4b. The Abstract, Methods, Results, and Discussion have also been revised consistently to emphasize that these outcomes represent database-captured adverse-event indicators within the indexed perioperative encounter rather than standardized 30-day postoperative complication rates.

Changes made:

• Methods, Section 3.3, “Outcome definitions”

• Supplementary Table S3

• Supplementary Tables S4a and S4b

• Corresponding clarifications in the Abstract, Results, and Discussion

Academic Editor Comment 2

“Procedure group classifications - 4. Is this appropriate enough. Please expand on it. But I do understand that this helps in statistical analysis.”

Response:

Thank you for this important comment. We agree that the rationale and construction of the four procedure groups required greater transparency. We have expanded the Methods to explain that this classification was developed as an analytical grouping for the MOVER EPIC cohort rather than as a validated surgical taxonomy or an ordinal measure of operative risk.

The source procedure categories were consolidated into four groups to preserve clinically interpretable differences in operative anatomy and general procedural complexity while avoiding excessively sparse categories in the regression models. The four groups were defined as follows:

1. Low-complexity common abdominal procedures, including appendectomy, cholecystectomy, and hernia repair;

2. Intestinal resection/stoma procedures, including colorectal resection, small-bowel resection, and stoma surgery;

3. Laparotomy/adhesiolysis, including open abdominal exploration and adhesiolysis-intensive procedures; and

4. Upper abdominal major surgery, including gastrectomy, pancreatic surgery, and splenic procedures.

Laparotomy/adhesiolysis was retained as a separate group because these procedures may reflect open exploration, complex reoperative surgery, or substantial adhesiolysis. Gastrectomy, pancreatic surgery, and splenic procedures were grouped as upper abdominal major surgery because they involve complex upper abdominal organ procedures and would otherwise have remained within a heterogeneous residual category.

We now explicitly state that these groups were intended to support clinically interpretable regression analyses and were not intended to imply uniform operative severity within each group. We have therefore interpreted procedure-group estimates cautiously, particularly because several procedure-group associations were attenuated after ASA adjustment or were sensitive to alternative model specifications.

The revised main manuscript now provides a brief description and representative procedures for each group. Supplementary Table S6 presents the complete mapping from the original procedure categories to the four analytical groups, and Supplementary Table S20 provides the clinical definitions and representative procedures. The reproducibility repository also contains the procedure-group mapping file used in cohort construction.

Changes made:

• Methods, Section 3.4, “Candidate predictors and covariates”

• Supplementary Table S6

• Supplementary Table S20

• Corresponding cautionary interpretation in the Results and Discussion

• Updated procedure-group mapping file in the reproducibility repository

Academic Editor Comment 3

“With small number of outcome events, provide more info on model stability and diagnostic assessments.”

Response:

Thank you for this important comment. We have expanded the statistical methods, results, and supplementary information to provide detailed model-stability and diagnostic assessments for both the primary broad-composite model and the secondary narrow-outcome model.

Both multivariable models contained eight estimated parameters, excluding the intercept. In the corrected primary complete-case cohort, the broad-composite model included 147 events, corresponding to 18.4 events per parameter. The narrow severe-composite model included 84 events, corresponding to 10.5 events per parameter.

We assessed sparse categorical cells, model convergence, complete and quasi-complete separation, and multicollinearity. No predictor category had zero events or zero non-events. The minimum number of events in a categorical level was 12 for the broad model and 3 for the narrow model. Maximum-likelihood estimation converged successfully for both outcomes, and neither complete nor quasi-complete separation was detected. The maximum variance inflation factor was 1.84, indicating no evidence of problematic multicollinearity.

Because the lower number of events, particularly in the narrow model, could increase small-sample bias in conventional maximum-likelihood estimates, we performed Firth penalized logistic regression for both outcomes. Both Firth models converged successfully, and the direction and magnitude of the principal associations remained broadly similar to the maximum-likelihood estimates. We also assessed the robustness of the primary model using patient-level cluster-robust standard errors, restriction to the first eligible encounter per patient, and inverse-probability weighting for complete-case selection.

We have added these diagnostic results to Supplementary Table S19. We also explicitly state that, although the narrow model met an events-per-parameter ratio of 10.5 and showed no evidence of separation or convergence failure, it contained fewer events and a minimum of only three events in one categorical level. Accordingly, the narrow-outcome analysis continues to be interpreted as supportive and exploratory rather than definitive.

Changes made:

• Methods, Section 3.7, “Statistical analysis”

• Results, primary model-stability assessment

• Results, secondary narrow-outcome analysis

• Supplementary Table S19

• Corresponding cautionary interpretation in the Discussion

Academic Editor Comment 4

“Provide more detail on excluded patients and its impact.”

Response:

Thank you for this important comment. We have expanded the description of the encounters excluded from the primary complete-case analysis and evaluated the potential impact of complete-case selection. Of the 5,679 encounters in the deduplicated adult analytic cohort, 330 (5.8%) were excluded from the primary regression cohort because of missing ASA physical status or operating-room duration. Among these encounters, 275 were missing both variables, 16 were missing ASA only, and 39 were missing operating-room duration only.

Compared with included encounters, excluded encounters had a higher frequency of the broad composite (5.76% versus 2.75%; p = 0.003), a longer median hospital length of stay (5 versus 3 days; p < 0.001), and a greater representation of laparotomy/adhesiolysis procedures (39.39% versus 21.95%; overall procedure-group p < 0.001). These differences suggest that complete-case selection may have under-represented encounters with greater clinical complexity or postoperative morbidity.

We therefore performed an inverse-probability-weighted sensitivity analysis. Among the 5,678 encounters with Female or Male sex coding, 5,349 were complete cases and 329 were non-complete cases. Stabilized weights showed good overlap, preserved nearly the full effective sample size, and improved measured covariate balance. The weighted estimates were materially similar to the primary complete-case estimates, although the confidence interval for ASA I–II included 1. We have therefore retained the primary complete-case analysis while adding a more cautious discussion of selection bias. We also acknowledge that IPW addresses selection conditional on measured variables and cannot eliminate bias related to unmeasured determinants of missingness.

Changes made:

• Methods, Section 3.7

• Results, comparison of included and excluded encounters

• Results, inverse-probability-weighted sensitivity analysis

• Discussion, limitations of complete-case selection

• Supplementary Tables S15 and S19

Reviewer #1

Comment:

“The study is methodologically sound, with the appropriate statistical analyses applied to both continuous and categorical variables. The use of descriptive statistics, regression analyses, and odds ratio is appropriate for the study objectives, and the conclusions are well supported by the presented results.”

Response:

We sincerely thank Reviewer #1 for the positive assessment of the methodological approach, statistical analyses, and interpretation of the findings. Although no specific revisions were requested by Reviewer #1, we have extensively revised the manuscript in response to the Academic Editor and Reviewer #2. These revisions include clearer outcome definitions, expanded documentation of the procedure-group classification, additional model diagnostics and sensitivity analyses, a detailed evaluation of complete-case selection, and more cautious interpretation of the EHR-derived outcomes and clinician-assigned ASA physical status.

Reviewer #2, Comment 1

“First, the outcome definitions require additional clarification. The primary and secondary outcomes are composites of EHR-captured adverse-event indicators, but these are not externally adjudicated complications and do not have a standardized 30-day follow-up window. The authors acknowledge this limitation, which is important. However, the manuscript would be stronger if the authors provided more explicit justification for each component included in the broad and narrow composites. For example, some broad categories may represent heterogeneous clinical events with very different severities.”

Response:

Thank you for this important observation. We agree that the components of the broad composite are clinically heterogeneous and should not be interpreted as events of uniform severity. We have therefore expanded the outcome definitions and added component-specific inclusion rationales.

The broad composite was constructed from the higher-level postoperative adverse-event categories available in MOVER EPIC, including cardiovascular, respiratory, airway, neurological, metabolic, injury/infection, and death-related events. These categories were retained to maximize the capture of postoperative morbidity signals recorded in the database. We now state explicitly that individual events within these categories may differ substantially in clinical severity and that the broad composite is neither a severity-graded endpoint nor an externally adjudicated complication measure.

The narrow composite was restricted to specific event-level indicators more directly representing major physiological deterioration or unplanned escalation of care. These included hemodynamic instability, hypotension, arrhythmia, cardiac arrest, hypoxemia, aspiration, hypercapnia, unplanned postoperative ventilation, reintubation, unplanned ICU admission recorded as a postoperative adverse-event item, and death. Administrative ICU indicators were excluded because they may represent planned postoperative monitoring or resource utilization rather than an adverse event.

Supplementary Table S4a now provides the clinical interpretation and explicit inclusion rationale for every component of both composites. Supplementary Table S4b reports the frequency of each component in the final adult analytic cohort. We also clarify that component counts are not mutually exclusive because one encounter could contain more than one recorded event, although each encounter contributed only once to the corresponding case-level composite.

Throughout the revised manuscript, we consistently describe these outcomes as “EHR-captured postoperative adverse-event indicator composites.” We emphasize that they reflect events captured within the indexed perioperative encounter and should not be interpreted as standardized 30-day postoperative complication rates or directly compared with registries using adjudicated definitions and standardized follow-up.

Changes made:

• Methods, Section 3.3, “Outcome definitions”

• Supplementary Table S3

• Supplementary Tables S4a and S4b

• Clarifying language in the Abstract, Results, and Discussion

• Expanded limitations concerning outcome heterogeneity, ascertainment, and the absence of standardized 30-day follow-up

Reviewer #2, Comment 2

“Second, the event rate is low. The broad composite occurred in 166 of 5,679 encounters, and the primary complete-case model included only 94 broad events. The narrow severe outcome included only 49 events in the complete-case cohort. The authors appropriately describe the narrow analysis as exploratory, but the low number of events limits the stability of the estimates, especially for categorical predictors with multiple levels. Please provide additional model diagnostics, such as the number of events per modeled parameter, assessment of sparse cells, convergence diagnostics, and whether penalized logistic regression was considered as a sensitivity analysis.”

Response:

Thank you for raising this important concern. As described in the “Important correction identified during revision” section of this response letter, the reproducibility audit identified that raw ASA codes 4 and 6 had been omitted from the earlier text-based ASA IV+ derivation. The overall adult cohort and outcome counts were unchanged, but correction of the ASA mapping increased the primary complete-case cohort from 4,798 to 5,349 encounters. Consequently, the number of broad-composite events in the primary model increased from 94 to 147, and the number of narrow severe-composite events increased from 49 to 84.

We nevertheless agree that the event counts, particularly for the narrow outcome, require explicit assessment of model stability. Both multivariable models contained eight estimated parameters, excluding the intercept. The corrected broad model therefore had 18.4 events per parameter, while the narrow model had 10.5 events per parameter.

We examined the categorical predictor levels for sparse event and non-event cells. No category had zero events or zero non-events. The minimum number of events in a categorical level was 12 for the broad outcome and 3 for the narrow outcome. Both maximum-likelihood models converged successfully, and neither complete nor quasi-complete separation was detected. The maximum variance inflation factor was 1.84, indicating no evidence of problematic multicollinearity.

In direct response to the reviewer’s suggestion, we performed Firth penalized logistic regression as a sensitivity analysis for both the broad and narrow outcomes. Both Firth models converged successfully. The direction and magnitude of the principal associations, particularly those involving clinician-assigned ASA physical status, remained broadly similar to the conventional maximum-likelihood estimates. We also performed patient-level cluster-robust inference and a first-encounter-per-patient sensitivity analysis to address repeated encounters.

These assessments are now reported in the Statistical Analysis and Results sections and summarized in Supplementary Table S19. Despite the successful convergence, absence of separation, and broadly consistent Firth estimates, we continue to describe the narrow-outcome model as supportive and exploratory because of its lower event count and the presence of a categorical level containing only three events.

Changes made:

• Updated all model-specific cohort and event counts

• Methods, Section 3.7, “Statistical analysis”

• Results, primary model-stability assessment

• Results, secondary narrow-outcome analysis

• Supplementary Tables S8b and S19

• Expanded cautionary interpretation of the narrow model

Reviewer #2, Comment 3

“Third, the complete-case approach may introduce selection bias. The manuscript states that excluded encounters had a higher frequency of captured broad adverse-event indicators, longer hospital stays, and a greater proportion of laparotomy/adhesiolysis procedures. This is important because it suggests that the primary complete-case cohort may under-represent higher-risk cases. The ASA-excluded sensitivity analysis is helpful, but the authors should expand the discussion of how this missingness could affect the direction and magnitude of the results. If feasible, multiple imputation or inverse-probability weighting could be considered as an additional sensitivity analysis. If not feasible, the rationale should be explained.”

Response:

Thank you. We agree that the higher broad-composite frequency, longer length of stay, and greater representation of laparotomy/adhesiolysis among excluded encounters required a more direct assessment of potential complete-case selection bias. We selected inverse probability weighting, one of the approaches suggested by the reviewer, because it allowed us to assess the sensitivity of the primary complete-case estimates to selection conditional on measured variables without imputing the missing ASA or operating-room-duration values. Among the 5,678 encounters with Female or Male sex coding, we modeled the probability of inclusion in the primary complete-case cohort using age, sex group, procedure group, the broad composite outcome, log-transformed hospital length of stay and log-transformed anesthesia duration, each with a missingness indicator, patient class, and ICU admission. We calculated stabilized weights and refitted the primary outcome model using sandwich-robust standard errors. The weights showed good overlap (mean 1.000, SD 0.035; range 0.949–1.178), no weight exceeded 1.25, and the effective sample size was 5,342.6. Measured covariate balance improved from a maximum absolute standardized mean difference of 0.024 before weighting to 0.001 after weighting. The IPW estimates were materially similar to the primary estimates for ASA IV+ (OR 4.78, 95% CI 3.06–7.48) and laparotomy/adhesiolysis (OR 1.81, 95% CI 1.02–3.19). The ASA I–II point estimate was essentially unchanged, but its robust confidence interval included 1 (OR 0.55, 95% CI 0.29–1.03), and we have softened the interpretation of this association accordingly. Results were also materially unchanged after truncation at the 1st and 99th percentiles. These additions are now reported in Methods Section 3.7, the Results model-stability paragraph, the Discussion limitations section, and Supplementary Table S19. We also note that IPW addresses selection conditional on measured variables and cannot exclude bias related to unmeasured determinants of missingness.

Changes made:

• Methods, Sections 3.6 and 3.7

• Results, comparison of included and excluded encounters

• Inverse-probability-weighted sensitivity analyses

• Supplementary Tables S15 and S19

• Expanded discussion of the possible direction and magnitude of complete-case selection bias

• Aggregate IPW estimates and weight diagnostics added to the reproducibility repository

Reviewer #2, Comment 4

“Fourth, the modeling strategy would benefit from more assessment of assumptions. Age and operating-room duration were modeled as continuous variables, but only operating-room duration appears to have been examined categorically in sensitivity analysis. Please consider assessing nonlinearity for both age and operating-room duration, for example using restricted cubic splines or clinically meaningful categories. In addition, because repeated encounters from the same patient were possible, the first-encounter sensitivity analysis is useful, but the authors should also consider clustered standard errors or a mixed-effects model if patient identifiers allow this.”

Response:

Thank you for these helpful suggestions. We have added analyses addressing both the functional-form assumptions for the continuous covariates and the potential within-patient correlation arising from repeated surgical encounters.

First, we assessed potential nonlinearity in age and operating-room duration using centered restricted cubic spline terms with three degrees of freedom. Each spline model was compared with the corresponding linear specification using a likelihood-ratio test. The age spline model showed modestly improved fit compared with the linear age specification (likelihood-ratio χ² = 6.39, df = 2; p = 0.041). The corresponding evidence for operating-room duration was weaker and did not reach statistical significance (χ² = 4.91, df = 2; p = 0.086). A model in which both age and operating-room duration were represented using centered spline terms also improved fit compared with the model containing both linear terms (χ² = 11.11, df = 4; p = 0.025).

We therefore revised the manuscript to acknowledge modest evidence of nonlinearity in the association between age and the broad composite. Importantly, the principal adjusted estimates remained materially unchanged under the alternative spline specifications. In the age-spline model, the adjusted OR was 4.79 (95% CI 3.00–7.67) for ASA IV+ versus ASA III, 0.55 (95% CI 0.30–0.99) for ASA I–II versus ASA III, and 1.79 (95% CI 1.03–3.14) for laparotomy/adhesiolysis versus low-complexity procedures. The corresponding estimates also remained similar when both continuous covariates were modeled using centered spline terms, as shown in Supplementary Table S19, Part E.

Because the purpose of these analyses was to assess sensitivity to the continuous-covariate functional form, and because the principal ASA and procedure-group estimates were materially unchanged, we retained the originally specified linear formulation as the primary parsimonious model and present the centered spline models as sensitivity analyses. However, we no longer state that the relationship with age was adequately characterized by a single linear term, and we caution against interpreting the per-year age odds ratio as a complete summary of the age–outcome relationship.

Second, because patient identifiers were available and some patients contributed more than one eligible encounter, we refitted the primary model using patient-level cluster-robust standard errors. This approach retained the surgical encounter as the analytical unit while accounting for within-patient correlation. The point estimates were unchanged, and the overall inference remained similar. ASA IV+ remained strongly associated with the broad composite, whereas the weaker laparotomy/adhesiolysis association became borderline under cluster-robust inference (OR 1.79, 95% CI 0.99–3.23; p = 0.053).

We also retained only the earliest eligible encounter for each patient and refitted the primary model. This first-encounter analysis included 4,704 encounters and 108 broad-composite events. ASA IV+ remained strongly associated with the outcome (OR 4.67, 95% CI 2.72–8.01), and the overall pattern of estimates remained consistent with the primary analysis.

We selected cluster-robust inference and restriction to the first eligible encounter as complementary approaches because the study objective concerned marginal encounter-level associations rather than estimation of patient-specific random effects. Together, these analyses addressed within-patient correlation without requiring assumptions concerning the distribution of a patient-level random intercept.

Changes made:

• Methods, Section 3.7, “Statistical analysis”

• Abstract and Results, interpretation of the age and operating-room-duration functional forms

• Supplementary Table S19, Parts D and E

• Results, patient-level cluster-robust and first-encounter analyses

• Supplementary Tables S16 and S17

Reviewer #2, Comment 5

“Fifth, the procedure-group classification should be described more transparently in the main manuscript. The grouping into low-complexity common abdominal procedures, intestinal resection/stoma, upper abdominal major surgery, and laparotomy/adhesiolysis is clinically reasonable, but the reader needs to understand what specific procedures were included in each group. The authors refer to supplementary tables, but a brief summary in the main text would improve interpretability.”

Response:

Thank you for this helpful suggestion. We agree that readers should be able to understand the four procedure groups from the main manuscript without relying exclusively on the supplementary information.

We have expanded the Methods to provide a brief definition and representative procedures for each group. Specifically:

1. Low-complexity common abdominal procedures included appendectomy, cholecystectomy, and hernia repair;

2. Intestinal resection/stoma procedures included colorectal resection, small-bowel resection, and stoma surgery;

3. Laparotomy/adhesiolysis included open abdominal exploration and adhesiolysis-intensive procedures; and

4. Upper abdominal major surgery included gastrectomy, pancreatic surgery, and splenic procedures.

We also clarify in the main manuscript that these groups were created to preserve clinically interpretable differences in operative anatomy and general complexity while avoiding sparse procedure categories in the regression models. The classification was intended as a study-specific analytical grouping rather than a validated surgical taxonomy or an ordinal severity scale.

Supplementary Table S6 provides the complete mapping from the original procedure categories to the four analytical groups. Supplementary Table S20 provides concise definitions and representative procedures, and the reproducibility repository includes the procedure-group mapping file applied during cohort construction.

Changes made:

• Expanded procedure-group description in Methods, Section 3.4

• Supplementary Table S6

• Supplementary Table S20

• Procedure-group mapping file in the reproducibility repository

Reviewer #2, Comment 6

“Sixth, the interpretation of ASA physical status should be cautious. ASA is a pragmatic clinical summary and is strongly associated with postoperative outcomes, but it is subjective and may incorporate clinician judgment, comorbidity burden, urgency, and other unmeasured risk factors. The manuscript appropriately notes this, but some parts of the discussion could overstate ASA as a direct marker of ‘baseline physiological vulnerability.’ I suggest consistently using language such as ‘clinician-assigned ASA physical status, which may reflect comorbidity burden and perceived perioperative vulnerability.’”

Response:

Thank you for this important clarification. We agree that clinician-assigned ASA physical status should not be interpreted as a direct or objective measurement of baseline physiological vulnerability, physiological reserve, or frailty.

We have revised the Abstract, Results, Discussion, and Conclusion to describe ASA consistently as a pragmatic, clinician-assigned clinical classification. We now state that it may reflect a combination of documented comorbidity burden, functional status, clinician judgment, perceived perioperative vulnerability, and other measured or unmeasured clinical information.

We have removed or revised wording that could imply that ASA directly measures physiological reserve or that the observed associations represent a causal effect of ASA classification. The revised Discussion emphasizes that ASA is partly subjective and may summarize clinical information that was not otherwise available as separate covariates in the MOVER EPIC dataset. Consequently, the ASA estimates may reflect residual confounding by underlying health status, comorbidity burden, functional limitation, urgency, or other unmeasured perioperative risk factors.

We have also revised our interpretation of the attenuation of procedure-group estimates after ASA adjustment. Rather than suggesting that ASA independently represents biological vulnerability, we now state that some operative-context information may overlap with the broader clinical risk context reflected in clinician-assigned ASA physical status.

The revised Conclusion therefore describes ASA as the most consistent correlate of the database-captured adverse-event indicators while explicitly stating that it should not be interpreted as a direct measure of physiological reserve or as an independently causal determinant of postoperative morbidity.

Changes made:

• Revised ASA terminology in the Abstract

• Revised interpretation of ASA in the Results and Discussion

• Removed wording implying direct measurement of physiological vulnerability or reserve

• Expanded discussion of subjectivity, clinician judgment, residual confounding, and unmeasured clinical information

• Revised Conclusion

Attachment Submitted filename: Response to the Academic Editor and Reviewers.docx

# Editor report: Decision Letter 1

**DOI:** 10.1371/journal.pone.0350704.r003

**Authors:** Jayaram Subramanian

8 Oct 2026

<p>Routinely available perioperative variables and EHR-captured postoperative adverse-event indicators after adult abdominal surgery: a retrospective cohort study using MOVER EPIC

PONE-D-26-24069R1

Dear Dr. Cui,

We’re pleased to inform you that your manuscript has been judged scientifically suitable for publication and will be formally accepted for publication once it meets all outstanding technical requirements.

Within one week, you’ll receive an e-mail detailing the required amendments. When these have been addressed, you’ll receive a formal acceptance letter and your manuscript will be scheduled for publication.

An invoice will be generated when your article is formally accepted. Please note, if your institution has a publishing partnership with PLOS and your article meets the relevant criteria, all or part of your publication costs will be covered. Please make sure your user information is up-to-date by logging into Editorial Manager at Editorial Manager® and clicking the ‘Update My Information' link at the top of the page. For questions related to billing, please contact billing support.

If your institution or institutions have a press office, please notify them about your upcoming paper to help maximize its impact. If they’ll be preparing press materials, please inform our press team as soon as possible -- no later than 48 hours after receiving the formal acceptance. Your manuscript will remain under strict press embargo until 2 pm Eastern Time on the date of publication. For more information, please contact onepress@plos.org.

Kind regards,

Jayaram Subramanian, Ph.D.

Academic Editor

PLOS One

Additional Editor Comments (optional):

Reviewers' comments:

# Editor report: Acceptance letter

**DOI:** 10.1371/journal.pone.0350704.r004

**Authors:** Jayaram Subramanian

PONE-D-26-24069R1

PLOS One

Dear Dr. Cui,

I'm pleased to inform you that your manuscript has been deemed suitable for publication in PLOS One. Congratulations! Your manuscript is now being handed over to our production team.

At this stage, our production department will prepare your paper for publication. This includes ensuring the following:

* All references, tables, and figures are properly cited

* All relevant supporting information is included in the manuscript submission,

* There are no issues that prevent the paper from being properly typeset

You will receive further instructions from the production team, including instructions on how to review your proof when it is ready. Please keep in mind that we are working through a large volume of accepted articles, so please give us a few days to review your paper and let you know the next and final steps.

Lastly, if your institution or institutions have a press office, please let them know about your upcoming paper now to help maximize its impact. If they'll be preparing press materials, please inform our press team within the next 48 hours. Your manuscript will remain under strict press embargo until 2 pm Eastern Time on the date of publication. For more information, please contact onepress@plos.org.

You will receive an invoice from PLOS for your publication fee after your manuscript has reached the completed accept phase. If you receive an email requesting payment before acceptance or for any other service, this may be a phishing scheme. Learn how to identify phishing emails and protect your accounts at https://explore.plos.org/phishing.

If we can help with anything else, please email us at customercare@plos.org.

Thank you for submitting your work to PLOS One and supporting open access.

Kind regards,

PLOS One Editorial Office Staff

on behalf of

Dr. Jayaram Subramanian

Academic Editor

PLOS One
